# Synthesis and Cytotoxicity Evaluation of Novel Coumarin–Palladium(II) Complexes against Human Cancer Cell Lines

**DOI:** 10.3390/ph16010049

**Published:** 2022-12-29

**Authors:** Edina H. Avdović, Marko Antonijević, Dušica Simijonović, Sunčica Roca, Dražen Vikić Topić, Nađa Grozdanić, Tatjana Stanojković, Ivana Radojević, Radiša Vojinović, Zoran Marković

**Affiliations:** 1 Department of Science, Institute for Information Technologies, University of Kragujevac, Jovana Cvijića bb, 34000 Kragujevac, Serbia; 2 NMR Centre, Ruđer Bošković Institute, Bijenička 54, 10000 Zagreb, Croatia; 3 Department of Natural and Health Sciences, Juraj Dobrila University of Pula, Zagrebačka 30, 52100 Pula, Croatia; 4 Department of Experimental Oncology, Institute of Oncology and Radiology of Serbia, Pasterova 14, 11000 Belgrade, Serbia; 5 Department of Biology and Ecology, Faculty of Science, University of Kragujevac, Radoja Domanovića 12, 34000 Kragujevac, Serbia; 6 Faculty of Medical Sciences, University of Kragujevac, Svetozara Markovića 69, 34000 Kragujevac, Serbia

**Keywords:** palladium(II) complexes, cytotoxicity, in silico, artificial intelligence, DFT optimization, molecular docking

## Abstract

Two newly synthesized coumarin–palladium(II) complexes (C1 and C2) were characterized using elemental analysis, spectroscopy (IR and ^1^H-^13^C NMR), and DFT methods at the B3LYP-D3BJ/6-311+G(d,p) level of theory. The in vitro and in silico cytotoxicity of coumarin ligands and their corresponding Pd(II) complexes was examined. For in vitro testing, five cell lines were selected, namely human cervical adenocarcinoma (HeLa), the melanoma cell line (FemX), epithelial lung carcinoma (A549), the somatic umbilical vein endothelial cell line (EA.hi926), and pancreatic ductal adenocarcinoma (Panc-1). In order to examine the in silico inhibitory potential and estimate inhibitory constants and binding energies, molecular docking studies were performed. The inhibitory activity of C1 and C2 was investigated towards epidermal growth factor receptor (EGFR), receptor tyrosine kinase (RTK), and B-cell lymphoma 2 (BCL-2). According to the results obtained from the molecular docking simulations, the inhibitory activity of the investigated complexes towards all the investigated proteins is equivalent or superior in comparison with current therapeutical options. Moreover, because of the low binding energies and the high correlation rate with experimentally obtained results, it was shown that, out of the three, the inhibition of RTK is the most probable mechanism of the cytotoxic activity of the investigated compounds.

## 1. Introduction

In the past decades, numerous studies have been carried out in an attempt to identify an effective drug or combinatorial therapy for various cancer types. The discovery of the anticancerogenic properties of cisplatin, back in 1965, was the basis for the invention of many metal-based compounds with a wide range of biological and physiological roles and activities. A substantial majority of these new metal-based compounds were determined to be unsuitable for clinical application due to excessive toxicity to healthy cells, interference with regular metabolic processes, easily established resistance of tumor cells to the potential medication, etc. Only a small number of compounds made their way to clinical usage. The anticancer drugs that are currently in use are insufficiently effective due to a variety of side effects, non-selectivity, resistance, and other factors. Therefore, new drugs that will be more effective in the fight against cancer are required [1,2,3].

Due to structural and thermodynamic similarities between platinum(II) and palladium(II) complexes, research is also focused on the synthesis of new Pd(II) complexes [4,5,6]. Numerous studies on palladium(II) complexes have been conducted, including those on their cytotoxicity and anticancer activity [4,5,6].

It is known that many plant-derived molecules act as antioxidants and have a synergistic effect when combined with other biologically active compounds. Hybrids obtained in this way often improve the biological properties of one or both parent molecules. This strategy could lead to the discovery of new compounds with promising therapeutic applications in the treatment of various cancers. Furthermore, the newly discovered compounds do not have to be new anticancer drugs; rather, they could be substances used to supplement existing therapeutic protocols [1,2,3].

Coumarins are an important group of natural compounds widely distributed in the natural kingdom [7]. Coumarin was originally isolated from the seeds of *D. odorata*, and after that it was isolated from numerous plants belonging to the families *Rutaceae, Umbelliferae* (*Apiaceae*), *Compositae* (*Asteraceae*), *Leguminosae*, and *Moraceae* [7]. In the plant world, they can be found in the highest concentration mainly in fruits and flowers, but also in seeds, roots, leaves, and stems [8]. Coumarins have a variety of pharmacological activities, including cytotoxic, antibacterial, and antifungal activity [9,10,11], and they are used to treat HIV [12,13], cancer [14,15], and neurological illnesses [16,17]. They were also employed for the scavenging of reactive free radical species [18,19]. Several reactive coumarin derivatives have already been produced, and their biological reactivity has been investigated and theoretically explained [20,21,22,23].

Coumarins, as biologically very active and therefore important compounds, were used to obtain Pd(II) complexes. Thanks to their lipophilicity and solubility, some palladium complexes have shown promising activity against various cell lines. For example, a palladium(II) complex with the tridentate ligand 3-(1-(2-hydroxyethylamino)ethylidene)chroman-2,4-dione was tested on several cell lines: L929 mouse fibrosarcoma, U251 human glioma, and B16 mouse melanoma. The IC_50_ values obtained indicate that this compound exhibits a greater cytostatic potency than the well-known cisplatin, inducing the apoptosis of cancer cells through caspase activation, mitochondrial depolarization, and oxidative stress [24]. In addition, Budzisz et al. synthesized an interesting coumarin–Pd complex that exhibits very high cytotoxicity against A549, HeLa, and K562 cells. This complex most likely attaches to double-stranded DNA as cisplatin induces changes in the short duplex DNA’s structure and lowers the electrophoretic mobility of plasmid DNA as a result. The activity of this compound is very similar to the activity of carboplatin and cisplatin [25].

Previous studies have shown that Pd(II) complexes containing coumarin hybrids have cytotoxic potential against pancreatic adenocarcinoma cell lines. [21,26]. This inspired the idea of developing new coumarin ligands and the investigation of malignant cancer cell lines. This research builds results that continue our prior work on the creation of novel coumarin-based ligands and corresponding palladium(II) complexes [20,27,28]. The obtained compounds were investigated for cytotoxicity in vitro and in silico. A molecular docking study was carried out to investigate the efficient binding of new compounds to receptor tyrosine kinase, as well as other proteins relevant for potential anticancer activity according to the literature and SwissTargetPrediction server. Additionally, the obtained compounds were tested in vitro for their antimicrobial activity.

## 2. Results and Discussion

### 2.1. Chemistry

**Scheme 1:**
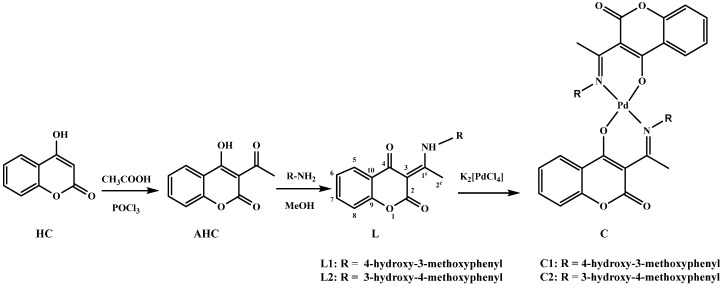
The general procedure for the synthesis of the ligands L1 and L2, and consequently the complexes C1 and C2.

The complexation reaction was performed by mixing equimolar amounts of ligands and potassium tetrachloropalladate(II) K_2_[PdCl_4_], resulting in the formation of palladium(II) complexes, C1 and C2 (Scheme 1). The stability of the investigated complexes was determined by UV-Vis spectroscopy. The investigated compounds excreted excellent stability during 48 h. The obtained UV-Vis spectra are presented in Figures S1 and S2 in the Supplementary Materials.

### 2.2. Spectroscopic and DFT Characterization

**Figure 1:**
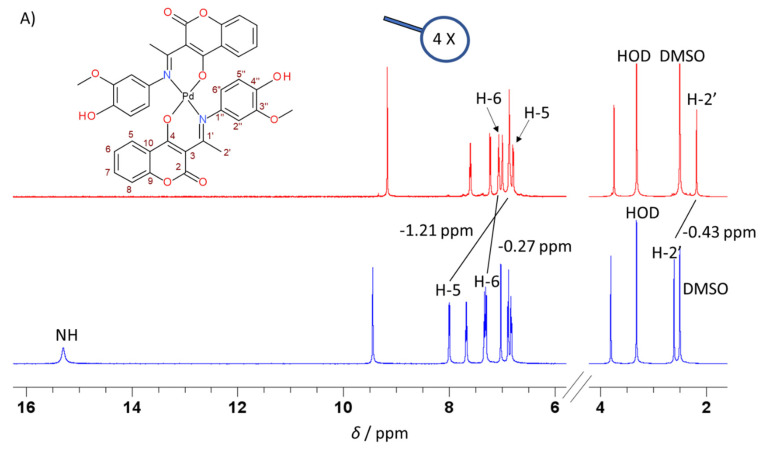

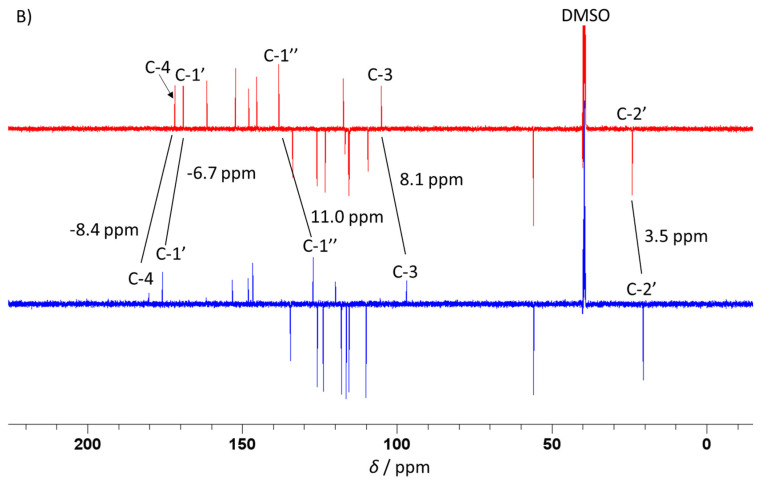
The main differences in the chemical shifts of the (**A**) ^1^H and (**B**) ^13^C atom signal after complexation between the ligand L1 (lower spectrum) and its Pd(II) complex C1 (upper spectrum). Coordination coefficients (∆δ_coord_.) are indicated to the lines in each case. The intensity in the downfield region of the 1H spectra (**A**) is multiplied by four.

One set of signals was obtained in the recorded ^1^H and ^13^C NMR spectra of the prepared Pd(II) complexes (C1, C2) in DMSO-d_6_. The coordination of the metal ion to the ligands [21] was confirmed by the absence of the NH group signal at 15.30 ppm in the ^1^H ligand NMR spectra and by the differences in the chemical shifts of the ^1^H and ^13^C atom signals in the NMR spectra after coordination (∆*δ*_coord._, complexation coefficient, calculated according to the relation ∆*δ*_coord._ = *δ*_complex_ − δ_ligand_). After binding to a metal ion, the signals of the H-5, H-2′ and H-6 atoms in the ^1^H NMR spectra were shielded (∆*δ*_coord._) by −1.21 ppm, −0.43 ppm, and −0.27 ppm, respectively (Figure 1A). All the other ^1^H atom signals of the complexes were negligibly shielded (∆*δ*_coord._ ≤ −0.1 ppm). The differences in the chemical shifts in the ^13^C NMR spectra provided more information about the coordination site of the Pd(II) ion (Figure 1B). The most significant changes were observed in the chemical shifts of the two shielded atom signals: C-4 and C-1′ (Δ*δ*_coord._ = −8.4 ppm, −6.7 ppm), and in the chemical shifts of the three deshielded atom signals: C-1′′, C-3, and C-2′ (Δ*δ*_coord._ = 11.0 ppm, 8.1 ppm, 3.5 ppm). The NMR spectroscopy results show that the ligands in the DMSO-d_6_ solution were bonded to the Pd(II) ion in a chelating mode via N,O-atoms. All recorded NMR spectra can be found in the Supplementary Materials (Figures S3–S8).

**Figure 2:**
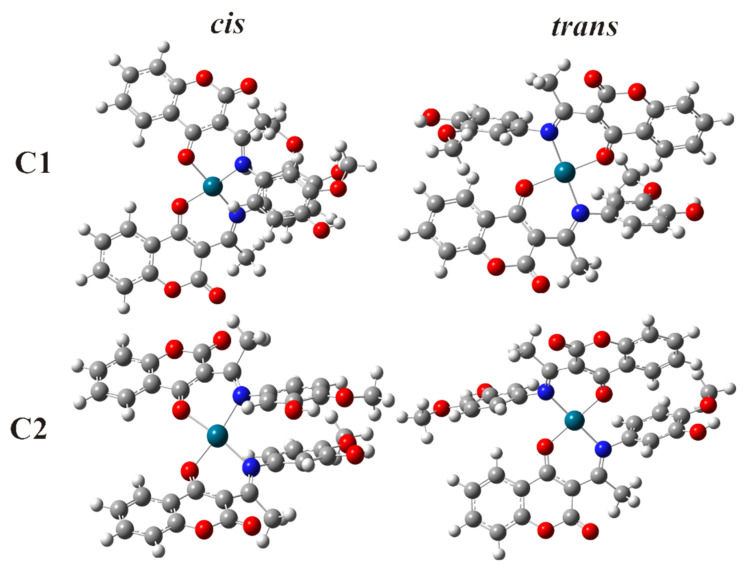
Optimized geometries of cis and trans isomers of C1 and C2.

Because X-ray crystallography could not be used to identify the dominant isomer of and overall structure of the investigated compounds, the DFT model mentioned in the methodology section was implemented. This theoretical model has been proven to be valid in previous studies of the structure of similar coumarin derivatives and their complexes [28,29]. The optimized structures of the cis and trans isomers of C1 and C2 are shown in Figure 2.

**Figure 3:**
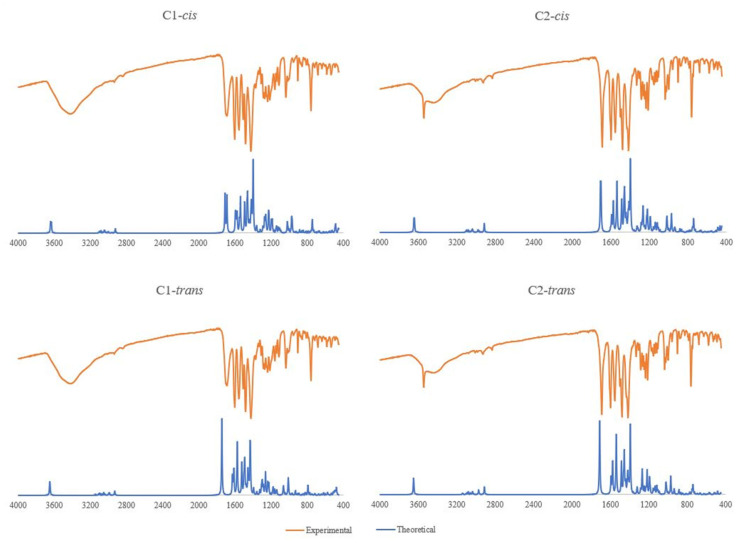
Experimental and theoretical IR spectra for C1 and C2.

Theoretically, two isomers, cis and trans, can be obtained in the last step of the synthesis process presented in Scheme 1. When the energies of these isomers were compared, it was revealed that the trans isomers were more stable in both circumstances. The energy differences between the two isomers are 11.26 and 16.11 kJ/mol for the C1 and C2 complexes, respectively, while the Boltzmann distribution values are 99.95% and 99.98%. Based on these numbers, the trans isomer is clearly dominant in both cases. This is due to the steric interference and charges repulsion of aromatic rings. Additionally, the NMR and IR spectra of both isomers were simulated to confirm that the hypothesized theoretical structures of the studied compounds correspond to the structure of the synthesized compounds. Tables S1–S3 show the structural parameters for the most stable structures of all studied compounds. Tables S4 and S5 provide the experimental and computed values for chemical shifts. The correlation coefficient (R) and mean absolute error (MAE) were used to assess the validity of the linear correlation between the experimental and computed chemical shifts in the NMR spectra. The relatively significant correlation coefficients for ^1^HNMR between 0.991 and 0.990, as well as the relatively small values of MAE, suggest that the computed geometries of examined compounds are in good correlation with the experimentally obtained structures [30,31,32]. When it comes to the cis isomer, correlation coefficients of 0.973 and 0.969 are a lot lower than for the trans isomer, which indicates that trans isomer is dominant, as predicted by Boltzmann distribution. As can be expected, simulated IR spectra for cis and trans isoforms are fairly similar, which can be seen in Figure 3. In addition, correlation coefficients were determined taking into account all the important peaks from the experimental data given in Section 3.1.2. More detailed information about the experimental and theoretical IR spectra is given in Table S6.

### 2.3. In Vitro Cytotoxicity

Parameters describing the cytotoxic activity of the investigated compounds, namely corresponding IC_50_ values, are presented in Table 1. Table 1 also includes the IC_50_ values for cisplatin as a positive control [33]. According to the obtained results, FemX cells were the most sensitive to the cytotoxic effect of the tested compounds. Moreover, the highest cytotoxic activity was exerted by the compound C1, followed by C2, on all tested cell lines. However, a closer examination of the results revealed that compounds C1 and C2 have very similar and consistent cytotoxic potential fairly similar to cisplatin. In comparison to cisplatin, C1 and C2 demonstrated better cytotoxic activity against A549 and Panc-1 cell lines, while cytotoxic activity against FemX was equivalent to cisplatin.

However, compounds L1 and L2 have less consistency and higher IC_50_ values, indicating lower cytotoxic activity than their corresponding Pd(II) complexes. Furthermore, when compared to cisplatin, ligands show significantly lower anticancer potential. Having this in mind, further analysis will be limited only to C1 and C2.

When the results of previous studies were compared, it was found that compounds with the -OH group showed significantly better cytotoxic potential than their analogues with chlorine substituents [21,28]. Furthermore, in regard to the results of the mentioned studies [21,28], the overall cytotoxic activity of C1 and C2 investigated in this paper was found to be rather good. However, since different cell lines were used when previous investigations were performed, direct comparison of IC_50_ values is most relevant when cell lines for pancreatic carcinoma (Panc-1 and MiaPaCa-2 [21]) are considered, since they are the most similar cell lines used. According to the results obtained in the previous research, the inhibitory activity of Pd(II) complexes with hydroxyl groups as substituents [21] was slightly higher (C1:IC_50_ = 3.0 and C2:IC_50_ = 6.0) than those with added methoxy groups (IC_50_ values 7.7 and 10.4, respectively) (Figure S9). The explanation for the disparity in IC_50_ values could lay in the fact that different cell lines of pancreatic carcinoma were used, and such comparison cannot give definitive conclusions.

### 2.4. Cell Cycle Analysis

**Figure 4:**
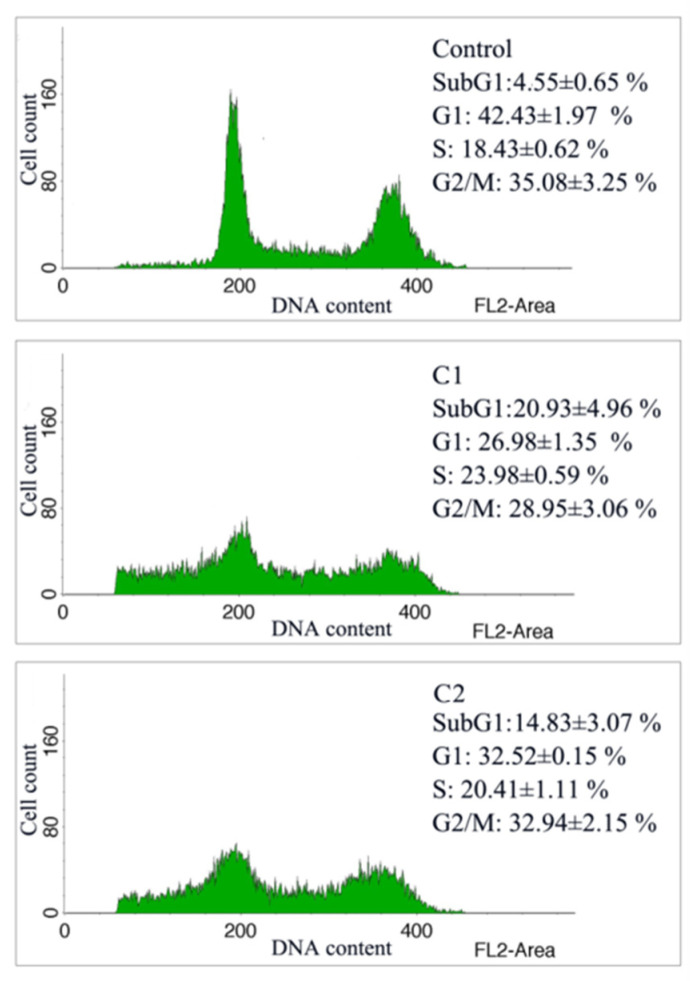
Cell cycle changes in phase distribution of HeLa cells induced by compound after 24 h of treatment.

**Figure 5:**
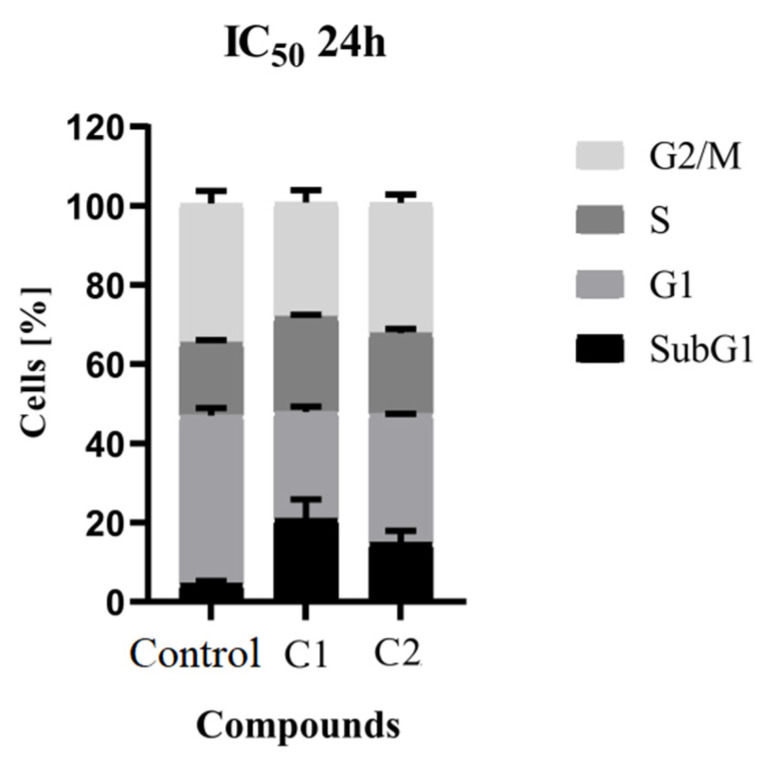
Cell cycle changes in phase distribution of HeLa cells induced by investigated compounds 24 h after treatment.

The compounds that showed the highest cytotoxic potential were further analyzed for the effect on HeLa cell cycle distribution, and the results are presented in Figure 4 and Figure 5. Analysis showed that compound C1 caused the highest increase in the proportion of HeLa cells in the SubG1 phase of the cell cycle when applied at the IC_50_ concentrations for 24 h (20.93 ± 4.96%, compared with control 4.55 ± 0.65%). Additionally, compound C2 showed a significant increase in the percentage of HeLa cells within the SubG1 phase of the cell cycle (14.83 ± 3.07%). An increase in the percentage of cells in the SubG1 phase was followed by a decrease in the number of cells in the G1 phase. These results indicate that, in vitro, compounds C1 and C2 exerted a significant proapoptotic effect on HeLa cancer cells.

### 2.5. Fluorescence Microscopy

**Figure 6:**
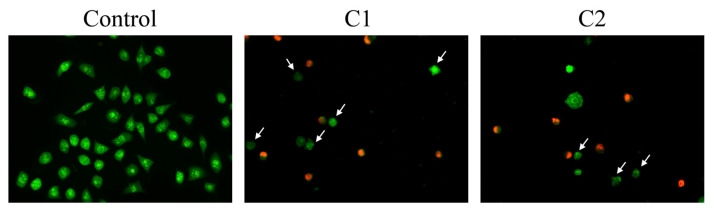
Representative fluorescence photomicrographs of acridine orange-ethidium bromide-stained HeLa cells 24 h after treatment with compounds C1 and C2 (detached cells from culture supernatant). Arrows are showing cells with typical signs of apoptosis (rounding, membrane blebbing, and condensed or fragmented nuclei).

After 24 h of exposure to the IC_50_ concentrations of compounds C1 and C2, the morphology of the HeLa cells was examined under a fluorescence microscope in order to verify the proapoptotic effect of the two tested compounds after cell cycle analysis (Figure 6). Because the majority of HeLa cells were found to be detached from coverslips following treatment, the supernatants were analyzed. The cells in the control well plates were still attached to the coverslips, with normal morphology and intact cell membranes, stained green. Both of the tested compounds caused a significant percentage of cells to exhibit the classic symptoms of apoptosis, including rounding, membrane blebbing, and condensed or fragmented nuclei. Moreover, on the photomicrographs, orange-red stained cells with damaged cell membranes were observed showing signs of late apoptosis (apoptotic bodies) and secondary necrosis.

### 2.6. In Vitro Scratch Assay

**Figure 7:**
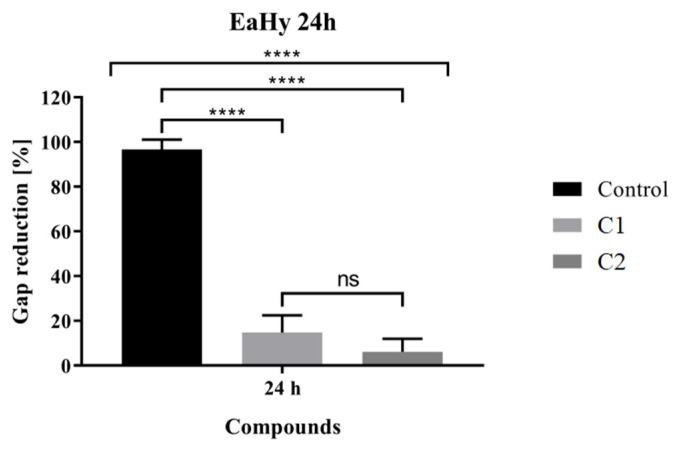
Scratch assay result chart showing percentages of gap reduction after 24 h treatments with IC_20_ concentrations of compounds C1 and C2 (**** *p* < 0.0001).

**Figure 8:**
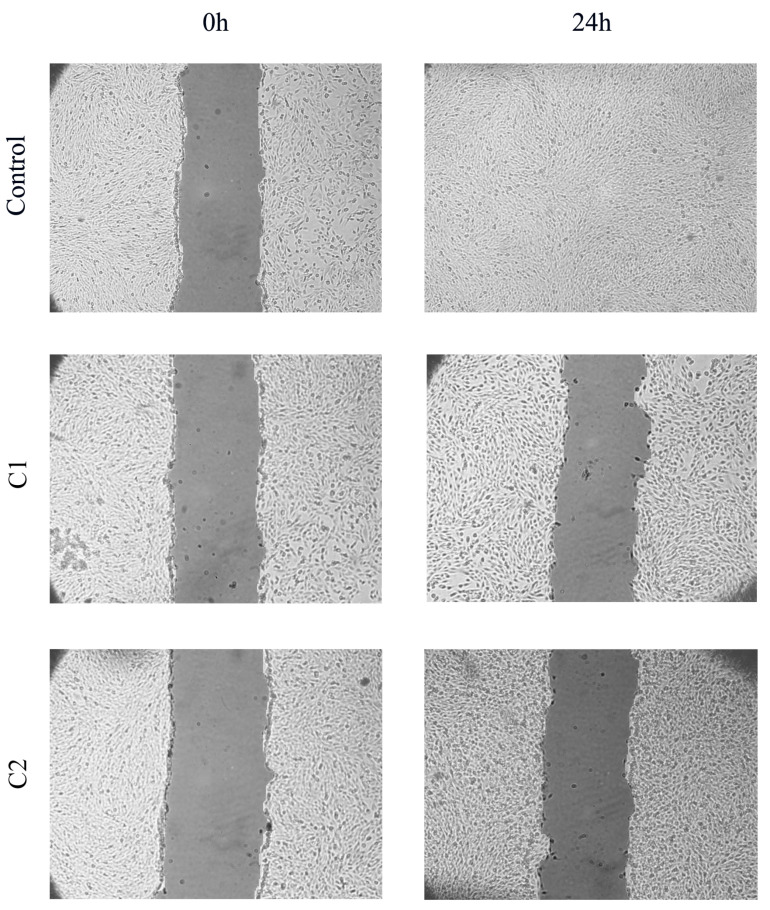
Representative photomicrographs of scratch assay before and after 24 h treatments of EA.hy926 cells with IC_20_ concentrations of compounds C1 and C2.

The effects of the two investigated compounds on the migration of endothelial EA.hy926 cells were assessed using an in vitro scratch assay (Figure 7 and Figure 8). Both compounds exerted significant antimigratory activities compared to the control (*p* < 0.0001). Compound C2 showed slightly better antimigratory activity (percentage of gap reduction was 6.14 ± 5.77), than C1 (14.78 ± 7.64), but the values of gap reduction were not significantly different.

### 2.7. Tube Formation Assay

**Figure 9:**
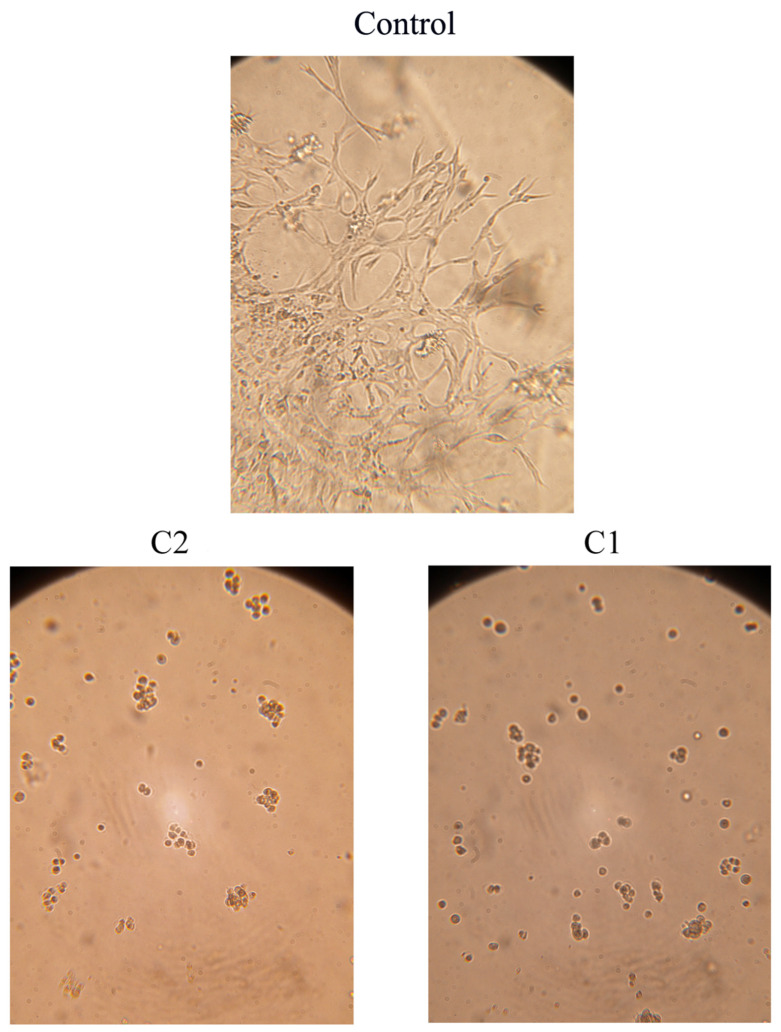
Representative photomicrographs of tube formation assay after 24 h treatments of EA.hy926 cells with IC_20_ concentrations of compounds C1 and C2.

Investigated Pd(II) complexes were examined for their in vitro antiangiogenic abilities by the utilization of the endothelial cell tube formation assay. Untreated control EA.hy926 cells formed large vessel-like structures and complex meshes. However, when EA.hy926 cells were treated with non-toxic IC_20_ concentrations of both compounds C1 and C2, after 24 h the cells appeared rounded and in clusters (Figure 9).

### 2.8. Molecular Docking Study

Potential anticancer agents can manifest their cytotoxic effect following a variety of distinct mechanistic pathways [21,31,34,35]. According to the results obtained from the SwissTargetPrediction webserver, the investigated Pd(II)-coumarin complexes are found to be possible inhibitors of EGFR and BCL-2. It is worth noting that similar compounds have been reported as potential inhibitors of RTK [19,35].

The inhibition of EGFR and RTK as part of the signal transduction pathway (STP) represents one of the newest and most promising approaches, when it comes to the development of new anticancer agents. It should be noted that EGFR is a protein that also belongs to the RTK enzyme family. However, because of its distinct structure, which is distinguished by asymmetric dimerization, it will be considered independently [34,35,36,37,38,39].

Another viable target for anticancer drug development is BCL-2, which is a crucial protein regulator of apoptosis. It is highly expressed in a variety of hematological malignancies and offers defense against cell death brought on by oncogenic and environmental stressors [40].

To test the inhibitory activity of L1, L2, C1, and C2 towards selected proteins, molecular docking simulations were performed, and the obtained results are presented in Table 2. Standards were used in order to compare the obtained results with compounds that are already in use. For the EGFR, *N*-(3-ethynylphenyl)-6,7-bis(2-methoxyethoxy)quinazolin-4-amine, also known under the commercial name Tarceva [41], was used as a standard. It is a chemotherapy drug that is used to treat non-small cell lung cancer or pancreatic cancer in a metastatic state. As a standard RTK inhibitor, 4-(2,4-dichloro-5-methoxyanilino)-6-methoxy-7-[3-(4-methylpiperazin-1-yl)propoxy]quinoline-3-carbonitrile, also known as Bosutinib, was used [42], while the BCL-2 inhibitor was an FDA-approved drug known as Navitoclax [43].

According to the results presented in Table 2, it is possible to see that the investigated complexes potentially inhibit all three of the investigated protein targets, which indicates multiple mechanistic pathways through which they can exhibit the cytotoxic effect. It is important to emphasize that, when it comes to C1 and C2, trans forms are taken into account, because simulations and experimental work showed that the trans forms of the investigated compounds are slightly more stable, and thus thermodynamically favored and prevalent.

It is interesting to notice that ligands L1 and L2 show similar inhibitory potential towards EGFR as C1 and C2. Similar binding energies obtained by molecular docking simulations are to be expected since both investigated complexes as well as ligand L1 occupy the same binding pocket and interact with the same amino acid residues (Figure S10). Only ligand L2 is found in the same active site as the standard, which indicates that only L2 can have comparable inhibitory potency towards EGFR. This is not in good correlation with experimentally obtained results, which indicates that EGFR inhibition is not a probable mechanism of the anticancer activity of the obtained Pd(II) coumarin complexes.

Another viable target for investigated compounds, according to the SwissADME webserver, BCL-2 is found on the outer membrane of mitochondria, where it promotes cellular survival and inhibits the effects of proapoptotic proteins. The BCL-2 family of proapoptotic proteins normally work on the mitochondrial membrane to induce permeabilization and the release of cytochrome C and ROS, which are crucial signals in the apoptosis cascade. The investigated compounds were found to inhibit BCL-2, which prevents it from fulfilling its original function, thus initiating, and inducing, apoptosis in the cancer cell. It was found that L2 and C1 show inhibitory potential similar to Navitoclax, while L1 and C2 show lower inhibitory potential when it comes to BCL-2 inhibition. The difference in binding energies between C1 and other investigated compounds is that C1 takes a slightly different conformation inside of the binding site, which allows it additional interactions with amino acids: HIS3, ALA4, and ARG6 (Figure S11). However, having in mind a correlation with the experimentally determined IC_50_ values and the inhibitory constants and binding energies obtained in molecular docking simulations of investigated compounds with BCL-2, we can conclude that inhibition of BCL-2 is also not a probable mechanistic pathway of the anticancer activity of the investigated compounds.

According to the results presented in Table 2, the lowest binding energies are found when it comes to the inhibition of RTK with C1 and C2. Furthermore, it should be emphasized that the best correlation of the results obtained by molecular docking simulations with experimental data is shown when it comes to the inhibition of RTK. These two facts indicate that the most probable mechanism of anticancer activity of C1 and C2, out of the three, is the inhibition of RTK, which is in accordance with the results obtained for similar compounds in our previous work [20,21,22].

It is interesting to notice that the ratio of the inhibitory constants for L1 and L2 is 2:1, which is nearly identical to the ratio of the IC_50_ values for the Panc-1 cell line. Moreover, the binding energies in Table 2 show that L1 and L2 have a moderate potential to inhibit RTK in comparison with the standard. However, experimental data indicate a slightly lower difference in the activity of L1 and L2 in comparison to C1 and C2, which may be a consequence of their low bioavailability or other pharmacokinetic characteristics of L1 and L2, which will be a subject of further investigations. It should be noted that inhibitory constants and IC_50_ for C1, C2, L1, and L2 also follow similar trends, when compared to one another, regarding the results obtained for the Panc-1 cell line. Furthermore, the data indicate that C1 shows a slightly better antagonistic effect than C2 towards the tested cell lines, demonstrating a good correlation between the experimental data and results obtained through molecular docking simulations.

In order to better explain the obtained results, a deeper look needs to be made into interactions that the investigated compounds form with RTK. AutoGridFR predicted that all the investigated compounds will show the lowest binding energies while occupying the Src homology 2 (SH2) domain, which in RTKs is characterized by tyrosine amino acid residues such as TYR463. The SH2 domain is found in many proteins involved in tyrosine kinase signaling cascades and their function is to bind tyrosine-phosphorylated sequences in specific protein targets. Moreover, the function of the SH2 domain is required for the tyrosine kinase to play a role in T-cell receptor signal transduction. SH2-domain-mediated interactions are an important step in receptor tyrosine kinase transmembrane signaling. Phosphatyrosine (pY) is recognized by SH2 domains in the context of certain sequence motifs in receptor phosphorylation sites. The inhibition of the SH2 domain of different proteins was previously reported as a promising drug design strategy against cancer, autoimmune diseases, and other diseases [44,45,46,47,48,49,50].

**Figure 10:**
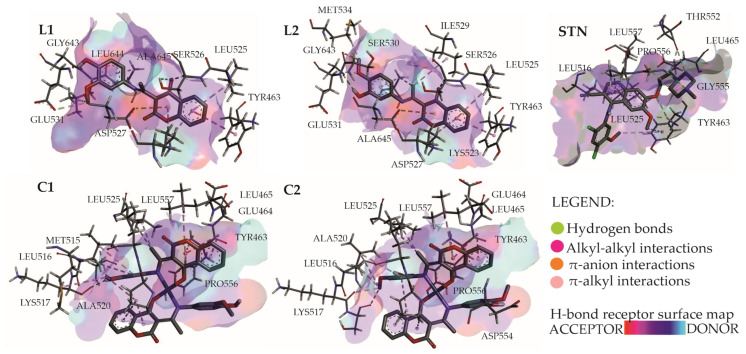
Molecular docking simulations: Interactions of L1, L2, C1, C2, and Bosutinib inside the active site of RTK, with H-bond receptor surface map.

As can be seen from Figure 10, all the investigated compounds were found to occupy the SH2 domain of RTK, defined by the following amino acids: TYR463, LEU557, LEU465, PRO556, LEU525, etc. The investigated compounds interact with a significant number of the same amino acid residues as Bosutinib, which is of importance since Bosutinib is a proven Src tyrosine kinase inhibitor [42].

A more streamlined representation of the most important interactions from Figure 10 is given in Table S7. As can be seen from Table S7, L1 and L2 do not form hydrogen bonds or electrostatic interactions with TYR residue, while C1, C2, and Bosutinib do. This is of special importance because tyrosine amino acid residues are responsible for the activity of the SH2 domain. In addition to the conventional hydrogen bonds with TYR463, C1 and C2 form strong electrostatic interactions with the same amino acid residues. Even though C2 forms one additional non-conventional hydrogen bond with TYR463, electrostatic interaction as well as the hydrogen bond formed with C1 is significantly shorter, which is reflected in binding energy, and consequently the activity of investigated compounds. Alongside hydrogen bonds and electrostatic interactions, ligands, as well as Bosutinib, form 6–7 hydrophobic interactions, while C1 and C2 form 12–15 hydrophobic interactions, all of which add to the final binding energies and stability of protein-substrate systems. Since these interactions are longer than 3.5Å, and to keep the simplicity of the presented results, the mentioned hydrophobic interactions are not presented in Table S7.

## 3. Materials and Methods

3-hydroxy-4-methoxy-aminophenol, 4-hydroxy-3-methoxy-aminophenol, 4-hydroxycoumarin, acetic acid, phosphoryl chloride, methanol, ethanol, toluene, acetone, and potassium tetrachloridopaladate(II) were purchased from Sigma Aldrich in Germany. A Vario EL III C, H, and N Elemental Analyzer was used to conduct the elemental analyses for the elements C, H, and N. A Perkin-Elmer Spectrum One FT-IR spectrometer was used to record infrared spectra (KBr) (4000–400 cm^–1^).

The NMR spectra were recorded on a Bruker AV600 spectrometer with a 5 mm probe head at the Ruđer Bošković Institute (Zagreb, Croatia). ^1^H and ^13^C APT NMR spectra were acquired at 600.130 and 150.903 MHz, respectively. Chemical shifts (*δ* / ppm) for the ^1^H and ^13^C spectra were referred to as the DMSO-d_6_ signals (^1^H: *δ* = 2.50 ppm; ^13^C: *δ* = 39.51 ppm), which were purchased from Euriso-Top, France. The assignments of ^1^H and ^13^C signals in the NMR spectra of compounds were confirmed by cross peaks obtained in the 2D spectra of ^1^H-^13^C Heteronuclear Multiple Bond Correlation (HMBC).

Silica gel (Silica gel 60, layer 0.20 mm, Alugram Sil G, Mashery-Nagel, Germany) was used for the analytical TLC, and a UV lamp (VL-4.LC, 365/254, Vilber Lourmat, France) was used for the visualization of TLC plates.

### 3.1. Chemical Studies

#### 3.1.1. Rationale for Choosing Ligand

Since palladium(II) complexes with coumarin hybrids with chlorine substituents [28] showed weaker activity towards cancer cell lines, chlorine was replaced by an OH group [21], which resulted in significantly better biological activity. A number of studies have shown that extracellular pH in tumors tends to be lower than that in normal tissue, and acidic pH promotes the invasive and metastatic potential of cancer cells [8,14]. In order to decrease the acidity of the compound, we introduced a methoxy group into the aromatic ring as an electron-donating group (EDW) in the ortho position relative to -OH. It is well known that EDWs reduce acidity because they push higher electron density towards a neighboring atom. Therefore, we expected that these compounds would show better activity than those previously investigated.

#### 3.1.2. General Procedure for Synthesis and Spectral Data of Palladium(II) Complexes

The synthesis of the bidentate coumarin ligands L1 and L2 has been previously reported [28,51]. To obtain their corresponding palladium(II) complexes C1 and C2, a methanolic solution of bidentate coumarin ligands (0.15 mmol) and an aqueous solution of potassium tetrachloropalladate(II) (0.15 mmol) were used. Solutions of ligands and palladium(II) salts were mixed with constant stirring on a magnetic stirrer at room temperature for 5 h. After precipitation, filtration, and air drying, the new palladium(II) complexes were obtained in moderately good yields (Scheme 1). The stability of the investigated complexes was confirmed by recording UV-Vis spectra in different timeframes, at 0 h, 24 h, and 48 h. Due to the good solubility of the investigated compounds in methanol, it was used as a solvent in which stability assessment experiments were performed (Figures S1 and S2).

*Bis(3-(1-((4–hydroxy-3-methoxyphenyl)amino)ethylidene) chromane-2,4–dione palladium(II)) complex (C1). Yellow powder. Yield: 0.037 g (69.81%). Anal. Calc. for* C_36_H_28_O_10_N_2_Pd (Mr = 754.08) %: C, 57.71; H, 4.06; N, 3.64. Found: C, 58.14; H, 3.95; N, 3.88. IR (KBr) ν cm^−1^: 3425 (OH); 2938 (=CH); 2843 (CH); 1690 (C=O); 1603 (C=N); 1557, 1483, and 1425 (C=C); 1036 (C–O); 536 (Pd–O); 452 (Pd–N). 1H NMR (DMSO-d6, 600.130 MHz): δH 9.17 (1H, br s, OH), 7.60 (1H, t, J = 7.53 Hz, H-7), 7.22 (1H, d, J = 8.11 Hz, H-8), 7.06 (1H, t, J = 7.53 Hz, H-6), 6.99 (1H, s, H-2′′), 6.89–6.81 (2H, m, H-5′′ and H-6′′), 6.79 (1H, d, J = 7.53 Hz, H 5), 3.74 (3H, s, OCH_3_), 2.18 (3H, s, H-2′) ppm. ^13^C NMR (DMSO-d6, 150.903 MHz): δC 171.7 (C-4), 169.0 (C-1′), 161.4 (C-2), 152.1 (C-9), 147.9 (C-3′′), 145.3 (C-4′′), 138.1 (C-1′′), 133.7 (C-7), 125.8 (C-5), 123.1 (C-6), 117.3 (C-10), 116.8 (C-8), 115.6 (C-5′′), 115.3 (C-6′′), 109.4 (C-2′′), 105.0 (C-3), 55.9 (OCH3), 24.0 (C-2′) ppm.

*Bis(3-(1-((3–hydroxy-4-methoxyphenyl)amino)ethylidene) chromane-2,4–dione palladium(II)) complex (C2). Yellow powder. Yield: 0.035 g (66.03%). Anal. Calc. for* C_36_H_28_O_10_N_2_Pd (Mr = 754.08)%; C, 57.71; H, 4.06; N, 3.64. Found: C, 58.09; H, 3.82; N, 3.78. IR (KBr) ν cm^−1^: 3437 (OH); 3010 (=CH); 2930, 2835 (CH); 1693 (C=O); 1601 (C=N); 1557, 1482, and 1419 (C=C); 1038 (C–O); 581 (Pd–O); 465 (Pd–N). ^1^H NMR (DMSO-d6, 600.130 MHz): δH 9.32 (1H, br s, OH), 7.59 (1H, td, J = 8.51, 1.51 Hz, H-7), 7.22 (1H, d, J = 8.33 Hz, H-8), 7.07 (1H, t, J = 7.85 Hz, H-6), 7.00 (1H, d, J = 8.57 Hz, H-5′′), 6.85–6.80 (2H, m, H-5 and H-2′′), 6.78 (1H, dd, J = 8.33, 2.14 Hz, H-6′′), 3.79 (3H, s, OCH3), 2.17 (3H, s, H-2′) ppm. ^13^C NMR (DMSO-d6, 150.903 MHz): δC 171.7 (C 4), 168.8 (C-1′), 161.3 (C-2), 152.1 (C-9), 147.0 (C 3′′), 146.4 (C-4′′), 139.5 (C-1′′), 133.6 (C-7), 126.0 (C-5), 123.1 (C-6), 117.2 (C-10), 115.5 (C-8), 115.0 (C-6′′), 112.2 (C-5′′), 112.1 (C-2′′), 104.9 (C-3), 55.78 (OCH3), 24.0 (C-2′) ppm.

#### 3.1.3. DFT Calculations

All calculations were carried out using the Gaussian16 program package, which augmented the Density Functional Theory (DFT) methods [52]. Structural geometries of investigated Pd(II)-coumarin complexes were obtained implementing the B3LYP-D3BJ method with the 6-311+G(d,p) basis set for C, N, O, and H, and LANLD2Z basis set for Pd(II) ions [53,54,55,56]. Optimization without any geometrical restraints produced the most stable structures of the examined compounds, and no imaginary frequencies were obtained, confirming that given conformations are found on the energy minima of the potential energy surface. The Gauge Independent Atomic Orbital (GIAO) method was utilized in order to simulate the ^1^H and ^13^CNMR spectra of the examined complexes [56,57]. The CPCM solvation model was used to investigate the probable solvent effects of DMSO [58]. To obtain values for the chemical shifts of the hydrogen and carbon atoms, values obtained for TMS, obtained under the same conditions as for C1 and C2, were subtracted from the corresponding values of the examined compounds. C1 and C2 complexes can exist in two states: cis and trans. It is well known that the Boltzmann distribution expresses the probability of a system being in each state as a function of its energy. The relative energy of the two conformers can thus be utilized to predict their distribution. The following equation is used for this purpose:

$$\frac{N_{cis}}{N_{trans}}=e^{-\left(E_{cis}-E_{trans}\right)/\mathrm{kT}}$$

where N*_cis_*, N*_trans_*, E*_cis_*, E*_trans_*, k, and T denote the number of particles in each state, the energy of both conformations, the Boltzmann constant, and temperature. Using the Boltzmann distribution to evaluate the ratio of two isomers was found to be effective in our previous work [28].

### 3.2. Biological Studies

#### 3.2.1. In Vitro Cytotoxicity

Cancer cell lines used in this study: human cervical adenocarcinoma (HeLa), melanoma cell line (FemX), human pancreatic adenocarcinoma (Panc-1), and lung epithelial carcinoma (A549) were maintained in complete RPMI-1640 medium. A somatic human umbilical vein endothelial cell line (EA.hy926) and pancreatic ductal adenocarcinoma (Panc-1) were grown in a DMEM medium. The cell lines were obtained from the American Type Culture Collection (USA). Cell lines A549, FemX, Panc-1, and EA.hy926 were seeded into 96-well microtiter plates. Seeding densities for each cell line were 5000 cells per well, except for the FemX cell line, with a seeding density of 3000 cells per well. Stock solutions of compounds (10 mM) were dissolved in dimethylsulfoxide (DMSO). The next day, cells were treated with serial dilutions of compounds ranging from 12.5 to 200 μM [59]. The final concentrations of DMSO applied to the cells were non-toxic and lower than 0.5%. Experiments were conducted in triplicate and repeated two times. An MTT cell survival assay was used to evaluate the effects of the compounds on cell survival after 72 h of treatment (for A549, FemX, and Panc-1) and 24 h treatment (for EA.hy926), described in detail [59]. IC_50_ was defined as the concentration of the compound that inhibited cell survival by 50% compared with the control.

#### 3.2.2. Cell Cycle Analysis

HeLa cells were seeded into 6-well plates (200,000 cells per well). After 24 h, they were incubated with the IC_50_ concentrations of investigated compounds for 24 h. The values of the IC_50_ concentrations applied were determined in advance and amount to 30.44 and 24.55 µM for C1 and C2, respectively. After incubation, the cells were trypsinized, collected, and fixed in 70% ethanol on ice and stored at −20 °C for 7 days [59]. Then, the cells were washed with PBS and incubated with RNaseA (100 μg/mL) for 30 min at 37 °C. After incubation with propidium iodide (40 μg/mL) for 10 min, cells were analyzed by a FACSCalibur flow cytometer (BD Biosciences Franklin Lakes, NJ, USA) using CELLQuest software. The results were obtained from two independent experiments and presented with standard deviations.

#### 3.2.3. Fluorescence Microscopy

Cancer HeLa cells were seeded into 6-well plates on the coverslips (50,000 cells per well). Compounds at IC_50_ concentrations were added to the cells after 24 h of adhesion. Cells were stained with a mixture of two dyes (3 μg/mL acridine orange and 10 μg/mL ethidium bromide in phosphate-buffered saline (PBS)) 24 h after the addition of the investigated compounds. Photomicrographs were taken under a fluorescence microscope, a Carl Zeiss PALM MicroBeam with AxioObserver.Z1 using AxioCamMRm (filters Alexa 488 and 568), as already described [59].

#### 3.2.4. In Vitro Scratch Assay

EA.hy926 cells were seeded into a 24-well plate (70,000 cells per well) in the complete DMEM medium. Confluent cell monolayers were formed after 24 h and scraped with a p200 pipette tip to create a straight central scratch line, as described earlier [59]. After washing with nutrient, medium cells were incubated with subtoxic concentrations (IC_20_) of investigated compounds for 24 h and the nutrient medium was added to the control wells. The IC_20_ concentrations of the compounds used are C1-2.75 μM and C2-3.50 μM. Photomicrographs were captured directly after making the wound and then 24 h later under the inverted phase-contrast microscope. The surface area of each wound was calculated using the open-source program ImageJ [60], at 0 h and after 24 h. Then, the percentages of the wound reduction for each well after 24 h were calculated. Results are shown as average values of three independent experiments and presented with standard deviations.

#### 3.2.5. Tube Formation Assay

The effects of the compounds on the inhibition of the angiogenesis of human endothelial EA.hy926 cells were analyzed by the endothelial cell tube formation assay [59].

Corning^®^ Matrigel^®^ Basement Membrane Matrix was gradually thawed overnight on ice, from −20 °C, where it was stored, to +4 °C. The 24-well plates and pipette tips were cooled to the same temperature (+4 °C). Then, 24-well plates (standing on ice) were coated with 200 μL of cold Corning^®^ Matrigel^®^ medium. The plates were then incubated for 2 h at 37 °C, in a CO_2_ incubator, in order to form a solid gel. EA.hy926 cells were then seeded into the wells (40,000 cells per well, suspended in 400 µL of complete DMEM medium). Only the nutrient medium was added to the control wells, while the others were treated with subtoxic (IC_20_) concentrations of investigated compounds. After 20 h of incubation, cells were observed and photographed under the inverted phase-contrast microscope.

#### 3.2.6. Antimicrobial Activity

The antimicrobial activity of the compounds was tested against 12 bacterial strains (4 standard strains, 3 clinical isolates, 5 isolates from nature) and 1 yeast species (Table S8). All clinical isolates were a generous gift from the Institute of Public Health in Kragujevac. The other microorganisms were provided from the collection of the Microbiology Laboratory of the Faculty of Science, University of Kragujevac (further details are given in the Supplementary Materials).

#### 3.2.7. Statistical Processing of the Information

All of the results obtained from in vitro studies were statistically processed by usage of a Student’s *t*-test. The *t*-test in statistics represents a method of testing hypotheses about the mean of a small sample drawn from a normally distributed population when the population standard deviation is unknown. Further information can be found in the following paper [61].

#### 3.2.8. Protocol of Molecular Docking Study

To investigate the inhibitory potential and evaluate inhibitory constants and binding energies, molecular docking studies were performed. In order to predict proteins that would be inhibition targets for the investigated compounds, the SwissTargetPrediction webserver, based on algorithms of artificial intelligence, was used [62]. The crystal structures of the proteins used in the molecular docking study were obtained from RCSB Protein Data Bank, with PDB IDs: 2KS1, 3GQL, and 1G5M, for epidermal growth factor receptors (EGFR), tyrosine kinase receptors (RTK), and B-cell lymphoma 2 (BCL-2), respectively [63]. Water molecules, cofactors, and co-crystalized ligands were deleted, and proteins were prepared for the simulation by using BIOVIA Discovery Studio 4.0 [64]. Ligands and complexes were prepared for simulations by geometry optimization using the Gaussian16 software package, implementing the B3LYP-D3 level of theory with Becke–Johnson empirical dispersion, and LANL2DZ/6-311+G(d,p), as mentioned in the Section 3.1.3. For performing molecular docking simulations, the AutoDock4.2 software package was used [65]. The Kollman partial charges and polar hydrogens were added using the AutoDockTools graphical interface. The flexibility of the ligands/complexes was taken into account, while the protein remained a rigid structure. For protein-complex flexible docking, the Lamarckian Genetic Algorithm (LGA) was used. The following parameters were determined for the LGA method: There were a maximum of 250,000 energy evaluations, 27,000 generations, and mutation and crossover rates of 0.02 and 0.8, respectively. AutoGridFR was utilized for the search of the active site and ligand orientation, and AutoDock 4.2.6 was implemented for the molecular docking energy calculations using Amber Force Field. The interactions between the target protein and the investigated compounds were analyzed and illustrated in 3D using BIOVIA Discovery Studio 4.0 and AutoDockTools.

## 4. Conclusions

In this study, two new palladium(II) complexes with bidentate coumarin-ligands have been synthesized, structurally characterized, and screened for cytotoxicity. In vitro cytotoxicity was tested on five different carcinoma cell lines: human cervical adenocarcinoma (HeLa), the melanoma cell line (FemX), lung epithelial carcinoma (A549), the somatic human umbilical vein endothelial cell line (EA.hy926), and pancreatic ductal adenocarcinoma (Panc-1). HeLa were the most sensitive to the cytotoxic effect of the tested compounds. The strongest cytotoxic activity on all the tested cell lines, except HeLa, was exerted by the C1 compound, followed by the C2 compound, while the coumarin ligands L1 and L2 had less potent cytotoxic effects. The compounds that showed the highest cytotoxic effect were further analyzed for the effect on HeLa cell cycle distribution. These results indicate that compounds C1 and C2 exerted a proapoptotic effect on HeLa cancer cells in vitro. To determine the mechanism of cytotoxic activity, molecular docking studies were employed. Proteins towards which inhibitory activity was investigated were selected according to the SwissTargetPrediction web server, as well as the available literature. It was found that the highest inhibitory activity was of C1 towards RTK, which is in excellent correlation to the experimental results. Moreover, it is worth mentioning that the investigated compounds show equivalent or superior inhibitory activity towards all three investigated proteins, in comparison with commercially available therapeutic standards.

## Figures and Tables

**Table 1:** IC_50_ values of the tested compounds and cisplatin.

IC_50_, µM *	FemX	A549	Panc-1	HeLa
L1	21.43 ± 1.59	78.83 ± 11.48	28.17 ± 7.40	>500
L2	27.56 ± 5.54	404.90 ± 6.94	72.71 ± 11.80	>500
C1	6.97 ± 0.36	10.7 ± 0.71	7.67 ± 0.74	5.68 ± 0.69
C2	7.67 ± 0.09	11.06 ± 0.41	10.43 ± 0.34	4.25 ± 0.85
Cis-Pt	6.16 ± 0.31	12.74 ± 1.26	16.44 ± 1.56	4.00 ± 0.47

* Concentrations of examined compounds inducing 50% decrease in cells’ survival rate (expressed as IC_50_ values). Results are presented as the mean value ± SD of the two independent experiments.

**Table 2:** Thermodynamic parameters obtained from molecular docking simulation for L1, L2, C1, C2, and standards with EGFR, RTK, and BCL-2.

	Protein	EGFR		RTK		BCL-2	
Ligand/Complex		ΔG_bind_	k_i_ (µM)	ΔG_bind_	k_i_ (µM)	ΔG_bind_	k_i_ (µM)
L1		−5.80	56.05	−8.80	0.35	−7.94	1.51
L2		−6.26	25.79	−8.39	0.70	−8.39	0.71
C1-trans		−6.47	18.09	−10.22	0.03	−8.56	0.53
C2-trans		−6.61	14.28	−9.40	0.13	−6.87	9.21
Standards		−4.93	240.00	−8.20	0.98	−8.69	0.43

## Data Availability

Data is contained within the article and Supplementary Materials.

## Supplementary Materials

The following supporting information can be downloaded at: https://www.mdpi.com/article/10.3390/ph16010049/s1, Figure S1. Stability assessment of C1 in different timeframes; Figure S2. Stability assessment of C2 in different timeframes; Figure S3. 600 MHz ^1^H NMR spectrum of C1 (DMSO-d6, 25 °C); Figure S4. 150 MHz ^13^C NMR spectrum of C1 (DMSO-d6, 25 °C); Figure S5. ^1^H-^13^C HMBC NMR spectrum of C1. The one-dimensional 600 MHz 1H spectrum is shown at the top edge, and a 150 MHz 13C NMR spectrum at the left-hand edge (DMSO-d6, 25 °C); Figure S6. 600 MHz ^1^H NMR spectrum of C2 (DMSO-d6, 25 °C); Figure S7. 150 MHz ^13^C NMR spectrum of C2 (DMSO-d6, 25 °C); Figure S8. ^1^H-^13^C HMBC NMR spectrum of C2. The one-dimensional 600 MHz ^1^H spectrum is shown at the top edge, and a 150 MHz ^13^C NMR spectrum at the left-hand edge (DMSO-d6, 25 °C); Figure S9. Structures of investigated complexes and IC_50_ values on pancreatic carcinoma cell lines (from previous work MiaPaCa-2 [21] (left) and from present investigation Panc-2 (right)); Figure S10. Molecular docking simulations: Interactions of L1, L2, C1, C2, and STN and EGFR, with H-bond receptor surface map; Figure S11. Molecular docking simulations: Interactions of L1, L2, C1, C2, and STN and BCL-2, with H-bond receptor surface map; Table S1. Bond lengths of the C1 and C2 in trans form; Table S2. Bond angles of the investigated compounds in the trans form; Table S3. Important dihedral angles of the investigated compounds in the trans form; Table S4. Experimental and calculated (by using the DFT/B3LYP-D3BJ method) chemical shifts (ppm) in the ^1^H NMR spectrum in DMSO for investigation compounds. The peak for the proton of the OH group was not considered due to the use of the implicit solvation model (CPCM); Table S5. Experimental and calculated (by using the DFT/B3LYP-D3BJ method) chemical shifts (ppm) in the ^13^C NMR spectrum in DMSO for investigated compounds; Table S6. Spectral data from the IR spectrums; Table S7. Interactions (conventional hydrogen bonds-CHB, non-conventional hydrogen bonds–NCHB, and electrostatic interactions-ESI) obtained from molecular docking simulations of inhibition of RTK with C1, C2, L1, L2 and STN. Lengths of formed interactions are given in Å; Table S8. Antimicrobial activity of tested compounds and positive controls. References [28,34,50,66,67,68] are cited in supplementary materials.
