# Supplementary material for: Synthesis and Cytotoxicity Evaluation of Novel Coumarin–Palladium(II) Complexes against Human Cancer Cell Lines
Source: Pharmaceuticals (Basel). 2022 Dec 29;16(1):49. doi: 10.3390/ph16010049 (PMC9866340; doi:10.3390/ph16010049)
Supplement: Supplementary file 1 [file pharmaceuticals-16-00049-s001.zip › pharmaceuticals-2083848-supplementary.pdf]

Synthesis and cytotoxicity evaluation of novel coumarin-palladium(II) complexes against human cancer cell lines

Edina H. Avdović[1*], Marko Antonijević[1], Dušica Simijonović[1], Sunčica Roca[2], Dražen Vikić Topić[2,3], Nađa Grozdanić[4], Tatjana Stanojković[4], Ivana Radojević[5], Radiša Vojinović[6], Zoran Marković[1]

[1]Institute for Information Technologies, Department of Science, University of Kragujevac, Jovana Cvijića bb, Kragujevac 34000, Serbia

[2]NMR Centre, Ruđer Bošković Institute, Bijenička 54, 10000 Zagreb, Croatia

[3]Department of Natural and Health Sciences, Juraj Dobrila University of Pula, Zagrebacka ˇ 30, Pula 52100, Croatia

[4]Institute of Oncology and Radiology of Serbia, Department of Experimental Oncology, Pasterova 14, 11000 Belgrade, Serbia

[5]Faculty of Science, Department of Biology and Ecology, University of Kragujevac, Radoja Domanovi´ca 12, 34000 Kragujevac, Serbia

[6]Faculty of Medical Sciences, University of Kragujevac, Svetozara Markovića 69, 34000 Kragujevac, Republic of Serbia

Correspondence: edina.avdovic@pmf.kg.ac.rs

Contents

Figure S1. Stability assessment of C1 in different timeframes.

Figure S2. Stability assessment of C2 in different timeframes.

Figure S3. 600 MHz ^{1}H NMR spectrum of C1 (DMSO-d6, 25 °C).

Figure S4. 150 MHz ^{13}C NMR spectrum of C1 (DMSO-d6, 25 °C).

Figure S5. ^{1}H-^{13}C HMBC NMR spectrum of C1. The one-dimensional 600 MHz 1H spectrum is shown at the top edge, and a 150 MHz ^{13}C NMR spectrum at the left-hand edge (DMSO-d6, 25 °C).

Figure S6. 600 MHz ^{1}H NMR spectrum of C2 (DMSO-d6, 25 °C).

Figure S7. 150 MHz ^{13}C NMR spectrum of C2 (DMSO-d6, 25 °C).

Figure S8. ^{1}H-^{13}C HMBC NMR spectrum of C2. The one-dimensional 600 MHz 1H spectrum is shown at the top edge, and a 150 MHz 13C NMR spectrum at the left-hand edge (DMSO-d6, 25 °C).

Figure S9. Structures of investigated complexes and IC_{50} values on pancreatic carcinoma cell lines (from previous work MiaPaCa-2 [21] (left) and from present investigation Panc-2 (right)).

Figure S10. Molecular docking simulations: Interactions of L1, L2, C1, C2, and STN and EGFR, with H-bond receptor surface map.

Figure S11. Molecular docking simulations: Interactions of L1, L2, C1, C2, and STN and BCL-2, with H-bond receptor surface map.

Table S1. Bond lengths of the C1 and C2 in trans form.

Table S2. Bond angles of the investigated compounds in the trans form.

Table S3. Important dihedral angles of the investigated compounds in the trans form.

Table S4. Experimental and calculated (by using the DFT/B3LYP-D3BJ method) chemical shifts (ppm) in the 1H NMR spectrum in DMSO for investigation compounds. The peak for the proton of the OH group was not considered due to the use of the implicit solvation model (CPCM).

Table S5. Experimental and calculated (by using the DFT/B3LYP-D3BJ method) chemical shifts (ppm) in the ^{13}C NMR spectrum in DMSO for investigated compounds.

Table S6. Spectral data from the IR spectrums

Table S7. Interactions (conventional hydrogen bonds - CHB, non-conventional hydrogen bonds – NCHB, and electrostatic interactions - ESI) obtained from molecular docking simulations of inhibition of RTK with C1, C2, L1, L2 and STN.Lengths of formed interactions are given in Å.

Table S8. Antimicrobial activity of tested compounds and positive controls

Biological studies

Materials and Methods

Antimicrobial activity

The antimicrobial activity of the compounds was tested against 12 bacterial strains (4 standard strains, 3 clinical isolates, 5 isolates from nature) and 1 yeast species (Table S8). All clinical isolates were a generous gift from the Institute of Public Health in Kragujevac. The other microorganisms were provided from the collection of the Microbiology Laboratory of the Faculty of Science, University of Kragujevac (details Supplemental Data section)

Bacterial suspensions were prepared by the direct colony method. The turbidity of the initial suspension was adjusted using a densitometer (DEN -1, BioSan, Latvia). Microbial inoculi were obtained from cultures incubated at 37°C for 24 h (bacteria) or 27°C for 48 h (yeast) and brought to approximately 108 CFU/mL or 106 CFU/mL by dilution according to the 0.5-McFarland standard [1]

Antimicrobial activity was tested by determining the minimum inhibitory concentration (MIC) and minimum microbicidal concentration (MMC) using the microdilution plate method with resazurin [1]. The 96-well plates were prepared by adding 100 µL of nutrient broth, Mueller-Hinton broth for bacteria and Sabouraud dextrose broth for yeast, to each well. The tested compounds were dissolved in DMSO and then diluted into the liquid culture medium to reach a concentration of 10%. An aliquot of 100 µl of the stock solution of the tested compound (with a concentration of 2000 µg/mL) was added to the first row of the plate. Then, a two-fold dilution series was performed using a multichannel pipette. The concentration range obtained was between 1000 and 7.8 µg/mL. The method is described in detail in the report [1].

Doxycycline and fluconazole were used as positive controls. It was found that 10% DMSO (as solvent control) did not inhibit the growth of microorganisms. Each test included a growth control and a sterility control. All tests were performed in duplicate, and MICs were constant. Minimum microbicidal concentrations were determined by plating 10 µL of samples from wells in which no indicator color formed onto nutrient agar. At the end of the incubation period, the lowest concentration with no colony was defined as the minimum microbicidal concentration.

Results and discussion

Antimicrobial activity

The results of *in vitro* antimicrobial activity of the tested compounds against 13 strains of microorganisms as well as the results of positive controls are presented in Table S8. The MIC and MMC values ranged from 31.25 µg/mL to > 1000 µg/mL.

The antimicrobial activity of the ligands and complexes tested was selective and moderate. Antimicrobial activity depends on the group or species of microorganisms. There is a difference in sensitivity between G- and G+ bacteria depending on the compounds tested. The most sensitive bacteria belong to the genus *Bacillus* (MIC 31.25/125 µg/mL for C1). At the same time, C1 among the substances shows higher activity than the corresponding ligand and the other complex C2.

Staphylococcus aureus ATCC 25923 and *Candida albicans* ATCC 10231 show no or weak sensitivity according to the tested concentrations (MICs and MMCs were 1000; > 1000 µg/mL). G bacteria are also resistant to the action of ligands and complexes. An exception is the effect of the C1 complex on the natural isolate of *Pantoea agglomerans* (Table S8).

In our previous studies on coumarin derivatives and their corresponding complexes, the intensity of antimicrobial activity also varied depending on the type of microorganism and the compound tested [1-3]. In most cases, palladium(II) complexes with a coumarin ligand showed lower antimicrobial activity

compared to commercial antibiotics and mostly no activity on gram-negative bacteria [1-4]. In some cases, the ligands showed better activity than the corresponding complexes and were active on bacteria of the *genus Bacillus* [1, 5, 6]. Similar to our study, some coumarin derivatives showed a broad spectrum of antimicrobial activity [4].

References

1. Avdović, E. H., Stojković, D. L., Jevtić, V. V., Milenković, D., Marković, Z. S., et. al. Preparation and antimicrobial activity of a new palladium (II) complexes with a coumarin-derived ligands. Crystal structures of the 3-(1-(o-toluidino) ethylidene)-chroman-2, 4-dione and 3-(1-(m-toluidino) ethylidene)-chroman-2, 4-dione. *Inorganica Chimica Acta*, 2019, 484, 52-59.
2. Avdović, E. H., Milanović, Ž. B., Živanović, M. N., Šeklić, D. S., Radojević, I. D., Čomić, L. R., et. al. Synthesis, spectroscopic characterization, biological activity, DFT and molecular docking study of novel 4-hydroxycoumarine derivatives and corresponding palladium (II) complexes. Inorganica Chimica Acta, 2020, 504, 119465.
3. Milanović, Ž. B., Marković, Z. S., Dimić, D. S., Klisurić, O. R., Radojević, I. D., Šeklić, D. S., et.al. Synthesis, structural characterization, biological activity and molecular docking study of 4, 7-dihydroxycoumarin modified by aminophenol derivatives. Comptes Rendus. Chimie, 2021, 24(2), 215-232.
4. Chin, Y. P., Huang, W. J., Hsu, F. L., Lin, Y. L., & Lin, M. H. Synthesis and evaluation of antibacterial activities of 5, 7-dihydroxycoumarin derivatives. Archiv der Pharmazie, 2011, 344(6) 386-393.
5. Ardakani, A. A., Kargar, H., Feizi, N., & Tahir, M. N. (2018). Synthesis, characterization, crystal structures and antibacterial activities of some Schiff bases with N_2O_2 donor sets. *Journal of the Iranian Chemical Society*, *15*(7), 1495-1504.
6. Arulmurugan, S., Kavitha, H. P., & Venkatraman, B. R. (2010). Biological activities of Schiff base and its complexes: a review. *Rasayan J Chem, 3*(3), 385-410.

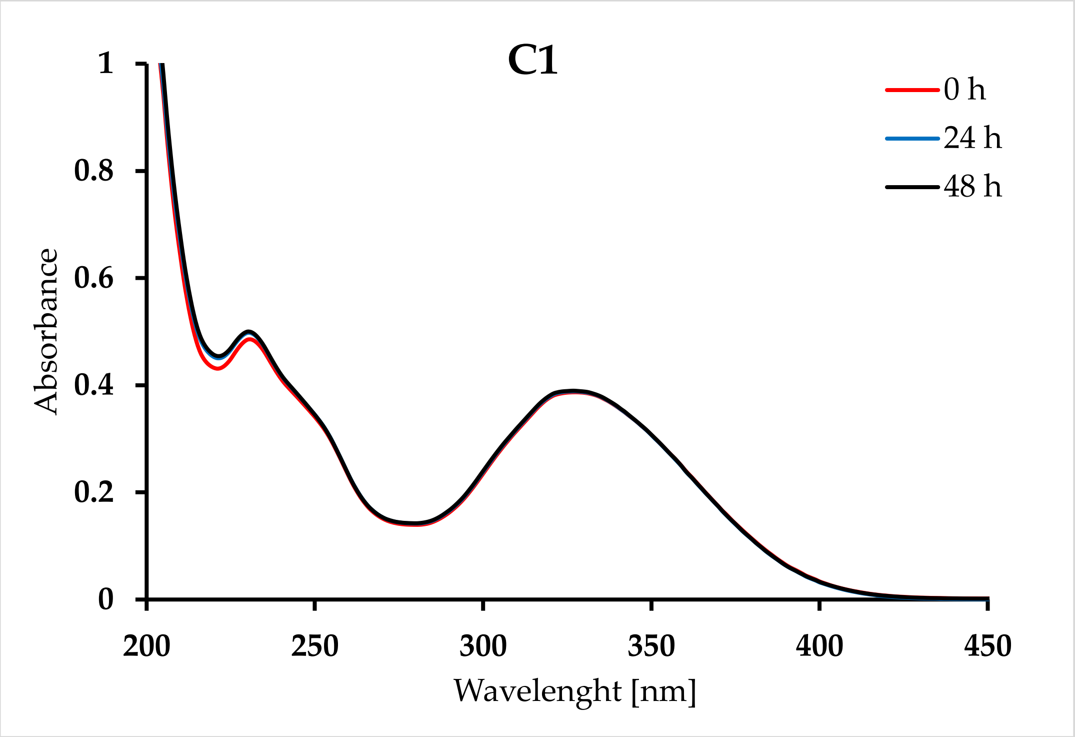

Figure S1. Stability assessment of C1 in different timeframes.

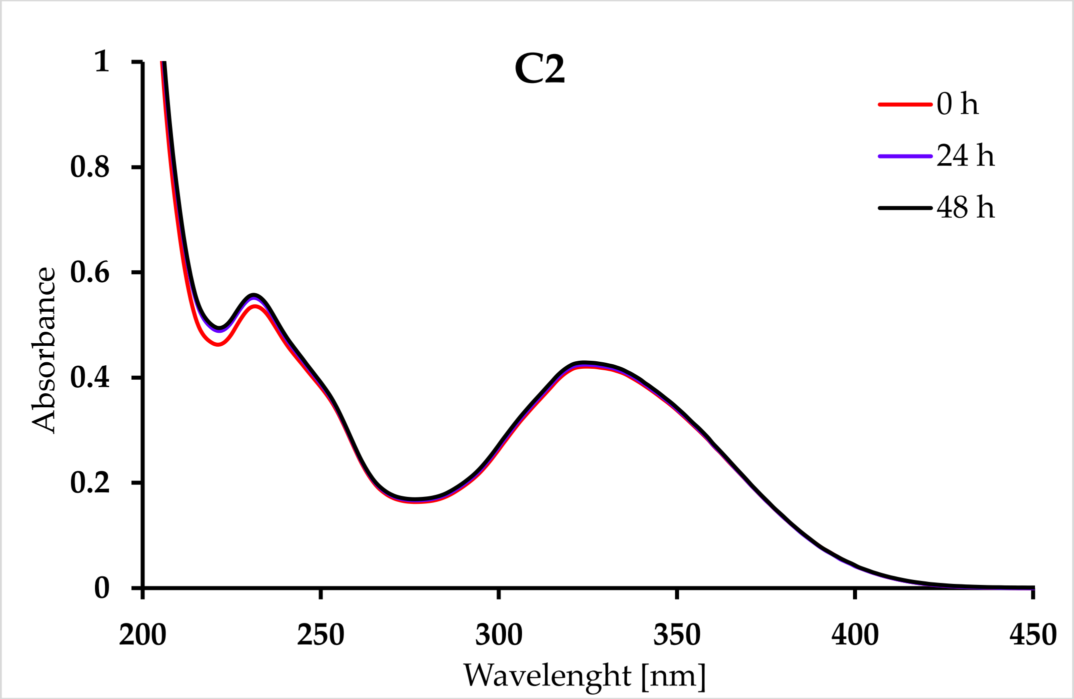

Figure S2. Stability assessment of C2 in different timeframes.

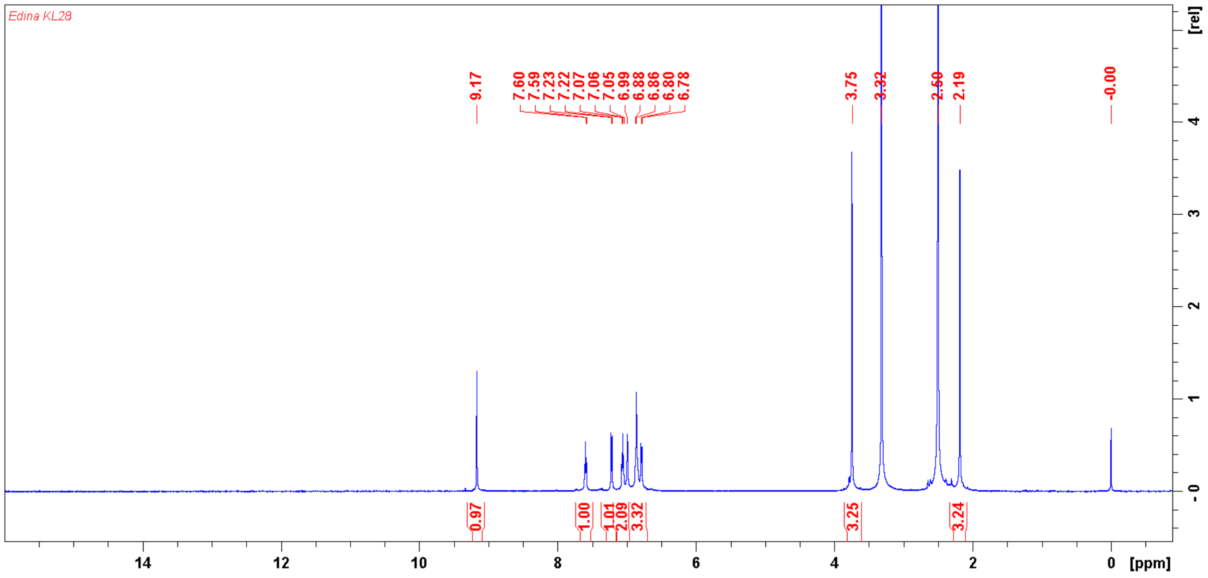

Figure S3. 600 MHz ^{1}H NMR spectrum of C1 (DMSO-d_6, 25 °C).

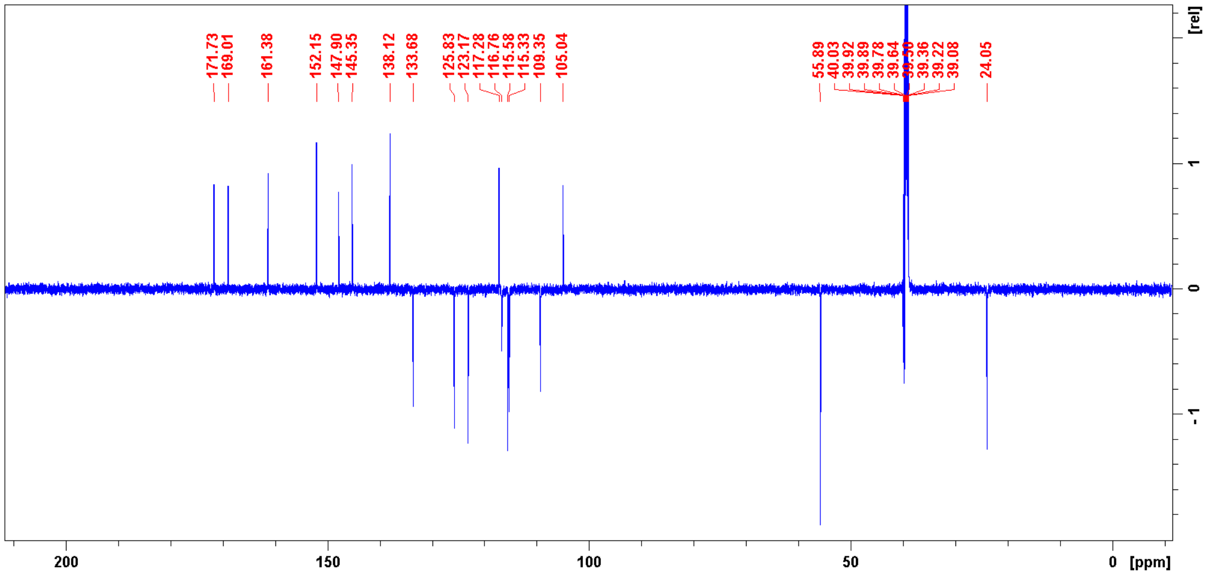

Figure S4. 150 MHz ^{13}C NMR spectrum of C1 (DMSO-d_6, 25 °C).

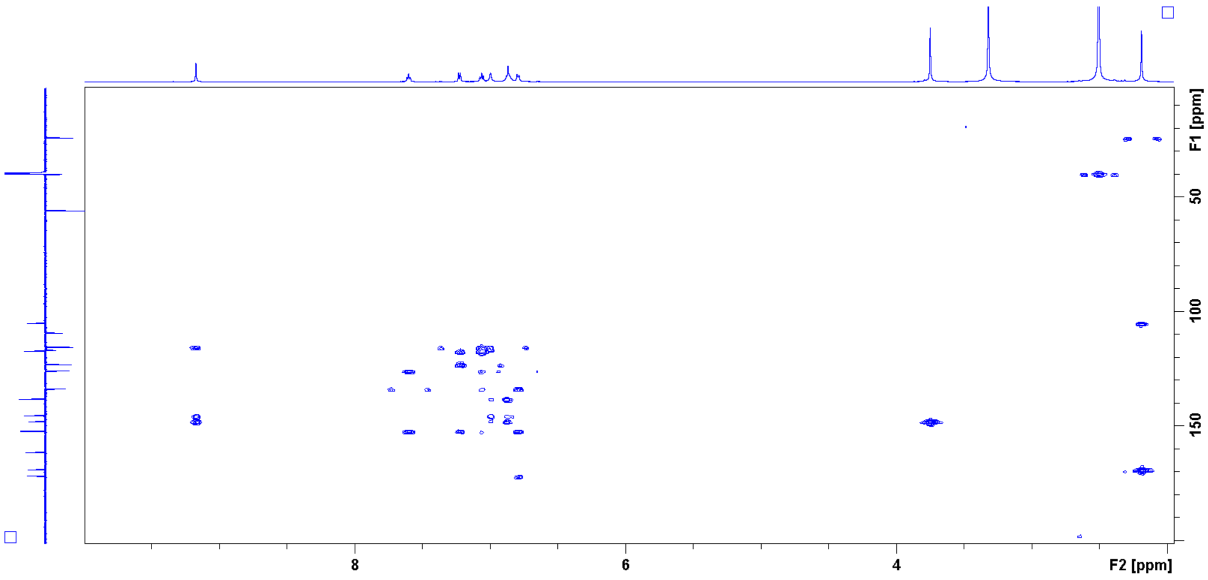

Figure S5. ^{1}H-^{13}C HMBC NMR spectrum of C1. The one-dimensional 600 MHz ^{1}H spectrum is shown at the top edge, and a 150 MHz ^{13}C NMR spectrum at the left-hand edge (DMSO-d_6, 25 °C).

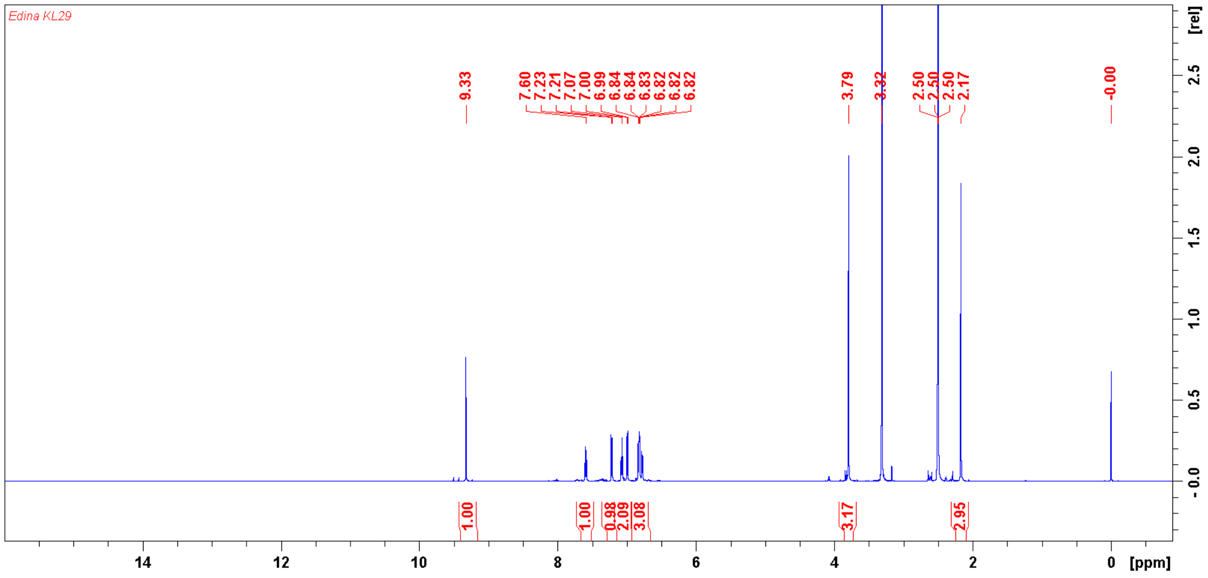

Figure S6. 600 MHz ^{1}H NMR spectrum of C2 (DMSO-d_6, 25 °C).

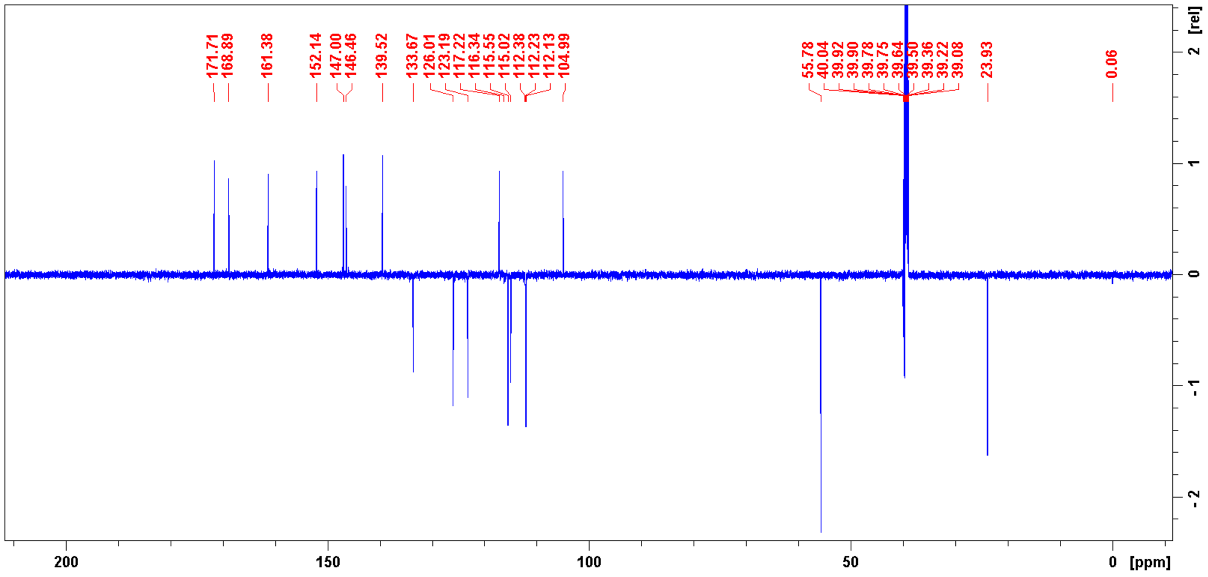

Figure S7. 150 MHz ^{13}C NMR spectrum of C2 (DMSO-d_6, 25 °C).

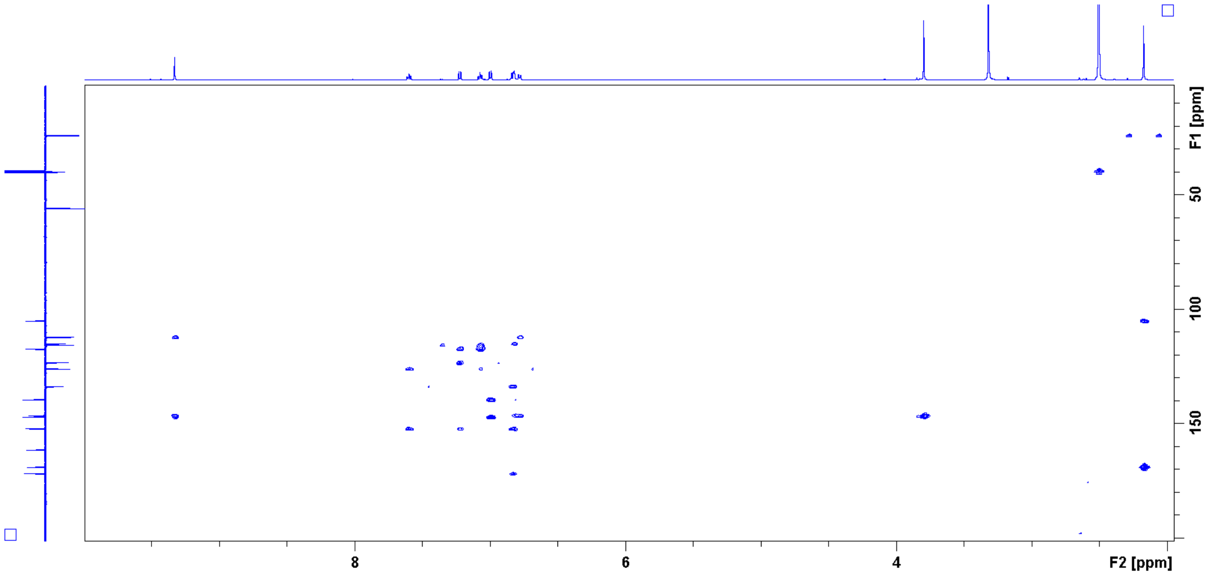

Figure S8. ^{1}H-^{13}C HMBC NMR spectrum of C2. The one-dimensional 600 MHz ^{1}H spectrum is shown at the top edge, and a 150 MHz ^{13}C NMR spectrum at the left-hand edge (DMSO-d_6, 25 °C).

Table S1. Bond lengths of the C1 and C2 in *trans* form.

Bond lengths (Å)	Calculated: DFT/B3LYP-D3BJ	
	**C1**	**C2**
**O1-C2**	1.40	1.40
**C2-C3**	1.46	1.46
**C3-C4**	1.41	1.41
**C4-C10**	1.46	1.46
**C10-C5**	1.40	1.40
**C5-C6**	1.38	1.38
**C6-C7**	1.40	1.40
**C7-C8**	1.39	1.39
**C8-C9**	1.39	1.39
**C9-C10**	1.39	1.39
**C9-O1**	1.36	1.36
**C3-C1’**	1.45	1.45
**C1’-C2’**	1.51	1.51
**C1’-N1’**	1.31	1.31
**N1’-C1”**	1.43	1.43
**C1”-C2”**	1.40	1.40
**C2”-C3”**	1.39	1.38
**C3”-C4”**	1.41	1.41
**C4”-C5”**	1.39	1.39
**C5”-C6”**	1.39	1.40
**C6”-C1”**	1.39	1.39
**C4”-OH**	1.36	/
**C3”-OH**	/	1.36
**C4”-OCH_3**	/	1.37
**C3”-OCH_3**	1.37	/
**C2=O**	1.21	1.21
**O4-Pd**	2.00	2.00
**N1’-Pd**	2.05	2.05

Table S2. Bond angles of the investigated compounds in the *trans* form.

Bond angles (º)<br>A-angle	Calculated: DFT/B3LYP-D3BJ	
	**C1**	**C2**
**A(C9–O1–C2)**	122.5	122.4
**A(O1–C9–C10)**	121.4	121.4
**A(O1–C2–C3)**	117.8	117.9
**A(O1–C2–O2)**	115.6	115.5
**A(C3–C2–O2)**	126.6	126.6
**A(C2–C3–C4)**	119.7	119.4
**A(C2–C3–C1')**	117.9	118.0
**A(C4–C3–C1')**	122.3	122.7
**A(C3–C4–C10)**	118.1	118.1
**A(C3–C4–O4)**	126.2	126.4
**A(C10–C4–O4)**	115.7	115.5
**A(C4–C10–C5)**	121.9	121.7
**A(C4–C10–C9)**	118.9	119.1
**A(C5–C10–C9)**	119.2	119.1
**A(C10–C5–C6)**	120.3	120.3
**A(C5–C6–C7)**	119.7	119.8
**A(C6–C7–C8)**	120.7	120.7
**A(C7–C8–C9)**	119.0	119.0
**A(C8–C9–C10)**	120.9	121.0
**A(C8–C9–O1)**	117.6	121.4
**A(C3–C1'–N1)**	122.7	122.7
**A(C3–C1'–C2')**	118.2	118.2
**A(N1–C1'–C2')**	118.0	118.9
**A(C1'–N1–C1")**	120.3	119.5
**A(N1– C1"–C2")**	118.9	119.4
**A(C1"–C2"–C3")**	119.5	119.9
**A(C2"–C3"–C4")**	120.4	119.9
**A(C3"–C4"–C5")**	119.5	120.0
**A(C4"–C5"–C6")**	119.9	119.9
**A(C5"–C6"– C1")**	120.0	119.9
**A(C6"-C1"-N1)**	120.7	120.1
**A(C2"–C3"–OH)**	/	119.8
**A(C2"–C3"– OCH_3)**	178.2	/
**A(OH–C3"–C4")**	/	120.2
**A(OCH_3–C3"–C4")**	114.1	/
**A(C3"–C4"–OCH_3)**	/	113.9
**A(C3"–C4"–OH)**	120.1	/
**A(OCH_3–C4"–C5")**	/	119.3
**A(OH–C4"–C5")**	120.2	/
**A(C4–OCH_3)**	/	118.1
**A(C3–OCH_3)**	118.1	/
**A(C1" –N1'–Pd)**	114.6	114.5
**A(N1'–Pd–O4)**	88.2	88.6
**A(C1' –N1'–Pd)**	125.1	125.9
**A(C4 –O4–Pd)**	123.4	125.2
**A(O4–Pd–O4)**	177.3	177.5
**A(N1'–Pd–N1')**	177.4	178.8

Table S3. Important dihedral angles of the investigated compounds in the *trans* form.

Bond angles (°)<br>DA-dihedral angles*	Calculated: DFT/B3LYP-D3BJ	
	**C1**	**C2**
**DA(C1'-C3-C4-O4)**	14.9	14.2
**DA(N1'-C1'-C3-C4)**	-24.3	-21.0
**DA(N1'-Pd-O4-C4)**	33.7	27.5
**DA(Pd-O4-C4-C10)**	161.7	167.0
**DA(Pd-O4-C4-C3)**	-20.5	-15.6
**DA(Pd-N1'-C1"-C5")**	109.1	-72.7
**DA(Pd-N1'-C1"-C2")**	-67.3	104.8
**DA(Pd-N1'-C1'-C3)**	2.6	2.2
**DA(O2-C2-C3-C1')**	13.5	15.3
**DA(O2-C2-C3-C4)**	-168.8	-166.3
**DA(C2-C3-C1'-C2')**	27.0	24.1
**DA(C2-C3-C1'-N1')**	-158.1	-160.7
**DA(C2'-C1'-N1'-C1")**	-4.9	-4.1
**DA(C3-C1'-N1'-C1")**	-179.25	-179.3
**DA(C1'-N1'-C1"-C2")**	114.7	-73.8
**DA(C1'-N1'-C1"-C5")**	-68.8	108.7
**DA(O1-C2-C3-C4)**	9.1	12.1
**DA(C2-C3-C4-C10)**	-14.8	-15.2
**DA(C2-C3-C4-O4)**	167.5	167.4
**DA(C5-C10-C4-O4)**	8.8	7.4
**DA(C9-C10-C4-O4)**	-171.5	-174.0
**DA(C4-C10-C5-C6)**	-179.4	-178.5
**DA(C10-C5-C6-C7)**	0.2	0.5
**DA(C5-C6-C7-C8)**	-0.7	-0.3
**DA(C6-C7-C8-C9)**	0.1	0.2
**DA(C7-C8-C9-C10)**	1.0	0.6
**DA(C7-C8-C9-O1)**	-180.0	-179.6
**DA(C1"-C2"-C3"-C4")**	-0.1	1.0
**DA(C2"-C3"-C4"-C5")**	-0.4	0.3
**DA(C3"-C4"-C5"-C6")**	-0.1	0.0
**DA(C4"-C5"-C6"-C1")**	1.1	-0.5
**DA(C5"-C6"-C1"-C2")**	-1.7	1.2
**DA(C6"-C1"-C2"-C3")**	1.2	1.5
**DA(C1"-C2"-C3"-OH)**	/	179.7
**DA(C2"-C3"-C4"-OCH_3)**	/	178.2
**DA(OH-C3"-C4"-OCH_3)**	/	0.5
**DA(C1"-C2"-C3"- OCH_3)**	-179.3	/
**DA(C2"-C3"-C4"-OH)**	-178.8	/
**DA(OH-C3"-C4"-OCH_3)**	0.5	/

Table S4.

Experimental and calculated (by using the DFT/B3LYP-D3BJ method) chemical shifts (ppm) in the ^{1}H NMR spectrum in DMSO for investigation compounds. The peak for the proton of the OH group was not considered due to the use of the implicit solvation model (CPCM).

^{1}H NMR Compound	Experimental		Theoretical			
	**C1**	**C2**	**C1**$_{cis}$	**C1**$_{trans}$	**C2**$_{cis}$	**C2**$_{trans}$
**-O-H**	9.17	9.32	/	/	/	/
**C7-H**	7.60	7.59	9.33	8.42	9.39	8.42
**C8-H**	7.22	7.22	8.44	7.91	8.59	7.97
**C6-H**	7.06	7.07	8.45	7.96	8.29	7.90
**C2”-H**	6.99	7.00	7.78	7.60	7.89	7.69
**C5”-H**	6.89-6.81	6.85-6.80	8.43	7.97	8.25	7.81
**C5**	6.79	6.78	6.85	7.03	6.66	6.93
**-OCH_3**	3.74	3.79	4.51	4.35	4.84	4.86
**C2’H_3**	2.18	2.17	3.00	2.94	2.68	2.86
**MAE**			**1.05**	**0.72**	**1.02**	**0.75**
**R**			**0.973**	**0.991**	**0.969**	**0.990**

Tabela S5. Experimental and calculated (by using the DFT/B3LYP-D3BJ method) chemical shifts (ppm) in the ^{13}C NMR spectrum in DMSO for investigated compounds.

^{13}C NMR Compound	Experimental		Calculated			
	**C1**	**C2**	**C1**$_{cis}$	**C1**$_{trans}$	**C2**$_{cis}$	**C2**$_{trans}$
**C1’**	169.0	168.8	175.7	177.3	175.7	177.4
**C4**	171.7	171.7	177.3	175.7	174.1	175.1
**C2**	161.4	161.3	164.9	164.9	165.0	164.8
**C9**	152.1	152.1	157.1	157.1	158.3	156.9
**C3”**	147.9	147.0	150.0	150.7	149.3	150.4
**C4”**	145.3	146.4	149.6	149.0	149.5	149.2
**C1”**	138.1	139.5	145.4	145.4	143.8	147.3
**C7**	133.7	133.6	136.7	136.7	136.9	136.7
**C5**	125.8	126.0	130.5	130.5	129.5	130.6
**C6**	123.1	123.1	125.1	125.1	125.8	124.6
**C6”**	115.3	115.0	122.2	120.7	121.8	119.9
**C10**	117.3	117.2	120.4	122.0	121.5	122.0
**C8**	116.8	115.5	117.6	117.6	118.6	117.7
**C5”**	115.6	112.2	115.2	115.2	111.2	111.7
**C2”**	109.4	112.1	111.2	111.2	115.8	114.9
**C3**	105	104.9	107.5	107.5	110.7	107.0
**-OCH_3**	55.9	55.78	54.2	54.2	54.2	54.0
**C2’**	24	24	25.9	25.9	25.6	26.5
	**MAE**		**3.3**	**3.3**	**3.4**	**3.4**
	**R**		**0.999**	**0.999**	**0.999**	**0.999**

Table S6. Spectral data from the IR spectrums

C1			C2		
Experimental	Theoretical		Experimental	Theoretical	
ν cm^{-1}	*cis*	*trans*	ν cm^{-1}	*cis*	*trans*
3425	3764	3726	3437	3781	3674
2938	3027	3020	3010	3070	3070
2843	3025	3015	2930	3018	3013
1690	1769	1722	2835	3017	3013
1603	1602	1602	1963	1769	1775
1557	1545	1543	1601	1598	1601
1483	1486	1484	1557	1543	1544
1425	1446	1410	1482	1489	1479
1036	1036	1037	1419	1401	1411
536	536	536	1038	1035	1038
452	452	451	581	581	579
R	0.998	0.999	465	466	467
			R	0.995	0.997

Table S7. Interactions (conventional hydrogen bonds - CHB, non-conventional hydrogen bonds – NCHB, and electrostatic interactions - ESI) obtained from molecular docking simulations of inhibition of RTK with C1, C2, L1, L2 and STN. Lengths of formed interactions are given in Å.

Interaction	L1	L2	C1	C2	STN
GLY643	1.94Å - CHB	2.23Å - CHB	/	/	/
SER526	2.99Å - CHB	2.12Å -NCHB	/	/	/
LYS523	1.98Å - CHB	/	/	/	/
SER526	2.02Å - NCHB	2.12Å – NHCB	/	/	/
GLU531	3.32Å - NCHB	2.42Å -NCHB	/	/	/
ASP527	4.32Å - ESI	/	/	/	/
SER530	/	3.11Å - NCHB	/	/	/
TYR463	/	/	4.34Å - ESI	4.52Å - ESI	1.98Å - CHB
LYS517	/	/	2.37Å - CHB	2.84Å - NCHB	/
TYR463	/	/	/	3.04Å -CHB	2.88Å - CHB
ASP554	/	/	/	2.02Å -NCHB	/
SER 518	/	/	/	3.33Å -NCHB	/
GLY555	/	/	/	/	2.62Å - CHB
LEU557	/	/	/	/	1.80Å - CHB
GLY555	/	/	/	/	3.23Å - NCHB
THR552	/	/	/	/	2.77Å - NCHB
THR552	/	/	/	/	3.09Å - NCHB

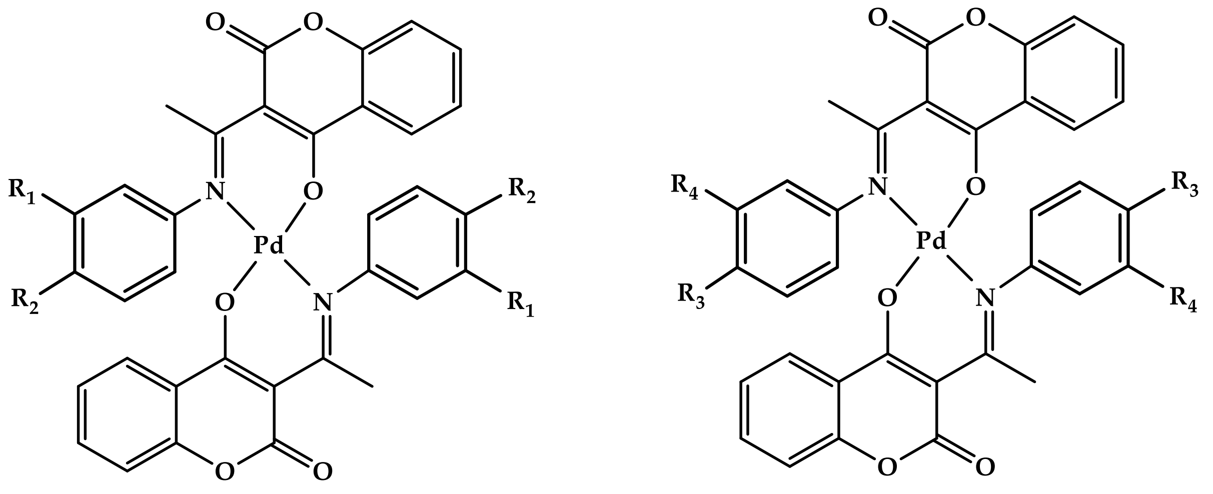

C1: R_1=OH; R_2=H; IC_{50}=3.0
C2: R_1=H; R_2=OH; IC_{50}=6.0

C1: R_3=OMe; R_4=OH; IC_{50}=7.7
C2: R_3=OH; R_4=OMe; IC_{50}=10.4

Figure S9. Structures of investigated complexes and IC_{50} values on pancreatic carcinoma cell lines (from previous work MiaPaCa-2 [21] (left) and from present investigation Panc-2 (right)).

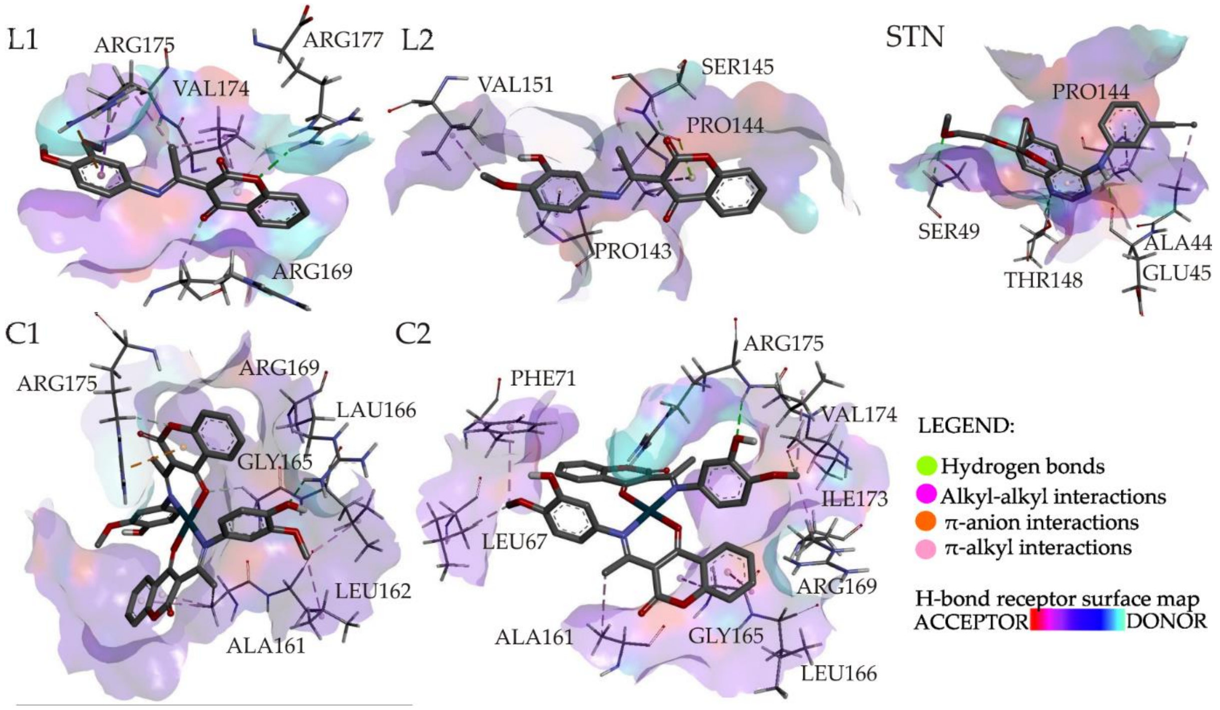

Figure S10. Molecular docking simulations: Interactions of L1, L2, C1, C2, and STN and EGFR, with H-bond receptor surface map.

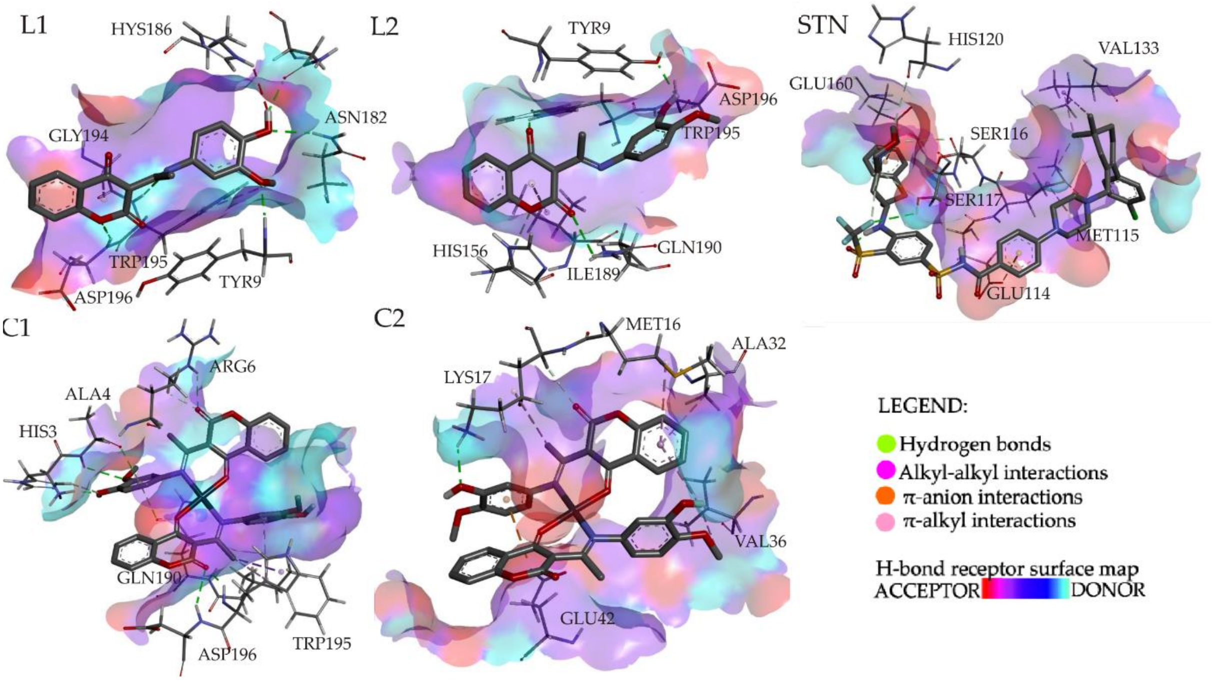

Figure S11. Molecular docking simulations: Interactions of L1, L2, C1, C2, and STN and BCL-2, with H-bond receptor surface map.

Table S8. Antimicrobial activity of tested compounds and positive controls

	L1		L2		C1		C2		Doxycycline / Fluconazole	
Species	MIC[1]	MMC[2]	MIC	MMC	MIC	MMC	MIC	MMC	MIC	MMC
Bacillus subtilis ATCC 6633	1000	1000	> 1000	> 1000	125	500	> 1000	> 1000	31.25	62.5
Bacillus altitudinis	500	1000	> 1000	> 1000	31.25	62.50	> 1000	> 1000	<0.5	<0.5
Bacillus pumilus	500	1000	> 1000	> 1000	31.25	1000	> 1000	> 1000	<0.5	<0.5
Bacillus cereus	1000	1000	> 1000	> 1000	125	1000	> 1000	> 1000	<0.5	3.91
Sarcina lutea	1000	1000	> 1000	> 1000	250	> 1000	1000	> 1000	<0.5	7.81
Staphylococcus aureus ATCC 25923	> 1000	> 1000	> 1000	> 1000	1000	> 1000	> 1000	> 1000	1.95	15.63
Pseudomonas aeruginosa ATCC 27853	1000	> 1000	1000	> 1000	1000	> 1000	1000	> 1000	7.812	62.5
Pseudomonas veronii	> 1000	> 1000	> 1000	> 1000	1000	> 1000	> 1000	> 1000	0.5	7.81
Proteus mirabilis ATCC 12453	> 1000	> 1000	> 1000	> 1000	> 1000	> 1000	> 1000	> 1000	125	125
Escherichia coli ATCC 25922	1000	1000	> 1000	> 1000	> 1000	> 1000	> 1000	> 1000	0.976	3.906
Escherichia coli	> 1000	> 1000	> 1000	> 1000	> 1000	> 1000	> 1000	> 1000	1.953	3.906
Pantoea agglomerans	1000	1000	1000	1000	31.25	125	1000	1000	0.5	7.81
Candida albicans ATCC 10231	1000	> 1000	> 1000	> 1000	> 1000	> 1000	> 1000	> 1000	0.5	1.95
